# Intracellular Accumulation of Novel and Clinically Used TB Drugs Potentiates Intracellular Synergy

**DOI:** 10.1128/Spectrum.00434-21

**Published:** 2021-09-29

**Authors:** Lloyd Tanner, Gabriel T. Mashabela, Charles C. Omollo, Timothy J. de Wet, Christopher J. Parkinson, Digby F. Warner, Richard K. Haynes, Lubbe Wiesner

**Affiliations:** a Division of Clinical Pharmacology, Department of Medicine, University of Cape Town, Cape Town, South Africa; b SAMRC/NHLS/UCT Molecular Mycobacteriology Research Unit, DST/NRF Centre of Excellence for Biomedical TB Research, Department of Pathology and Institute of Infectious Disease and Molecular Medicine, Faculty of Health Sciences, University of Cape Town, Cape Town, South Africa; c School of Biomedical Sciences, Charles Sturt University, Orange, New South Wales, Australia; d Wellcome Centre for Infectious Diseases Research in Africa, University of Cape Town, Cape Town, South Africa; e Centre of Excellence for Pharmaceutical Sciences, Faculty of Health Sciences, North-West University, Potchefstroom, South Africa; Johns Hopkins University School of Medicine

**Keywords:** TB chemotherapy, intracellular accumulation, macrophages, intracellular efficacy, phenoxazine, artemisinin, synergism tuberculosis

## Abstract

The therapeutic repertoire for tuberculosis (TB) remains limited despite the existence of many TB drugs that are highly active in *in vitro* models and possess clinical utility. Underlying the lack of efficacy *in vivo* is the inability of TB drugs to penetrate microenvironments inhabited by the causative agent, Mycobacterium tuberculosis, including host alveolar macrophages. Here, we determined the ability of the phenoxazine PhX1 previously shown to be active against M. tuberculosis *in vitro* to differentially penetrate murine compartments, including plasma, epithelial lining fluid, and isolated epithelial lining fluid cells. We also investigated the extent of permeation into uninfected and M. tuberculosis-infected human macrophage-like Tamm-Horsfall protein 1 (THP-1) cells directly and by comparing to results obtained *in vitro* in synergy assays. Our data indicate that PhX1 (4,750 ± 127.2 ng/ml) penetrates more effectively into THP-1 cells than do the clinically used anti-TB agents, rifampin (3,050 ± 62.9 ng/ml), moxifloxacin (3,374 ± 48.7 ng/ml), bedaquiline (4,410 ± 190.9 ng/ml), and linezolid (770 ± 14.1 ng/ml). Compound efficacy in infected cells correlated with intracellular accumulation, reinforcing the perceived importance of intracellular penetration as a key drug property. Moreover, we detected synergies deriving from redox-stimulatory combinations of PhX1 or clofazimine with the novel prenylated amino-artemisinin WHN296. Finally, we used compound synergies to elucidate the relationship between compound intracellular accumulation and efficacy, with PhX1/WHN296 synergy levels shown to predict drug efficacy. Collectively, our data support the utility of the applied assays in identifying *in vitro* active compounds with the potential for clinical development.

**IMPORTANCE** This study addresses the development of novel therapeutic compounds for the eventual treatment of drug-resistant tuberculosis. Tuberculosis continues to progress, with cases of Mycobacterium tuberculosis (M. tuberculosis) resistance to first-line medications increasing. We assess new combinations of drugs with both oxidant and redox properties coupled with a third partner drug, with the focus here being on the potentiation of M. tuberculosis-active combinations of compounds in the intracellular macrophage environment. Thus, we determined the ability of the phenoxazine PhX1, previously shown to be active against M. tuberculosis *in vitro*, to differentially penetrate murine compartments, including plasma, epithelial lining fluid, and isolated epithelial lining fluid cells. In addition, the extent of permeation into human macrophage-like THP-1 cells and H37Rv-infected THP-1 cells was measured via mass spectrometry and compared to *in vitro* two-dimensional synergy and subsequent intracellular efficacy. Collectively, our data indicate that development of new drugs will be facilitated using the methods described herein.

## INTRODUCTION

Tuberculosis (TB) remains a leading cause of death from an infectious disease, surpassing both malaria and human immunodeficiency virus/acquired-immunodeficiency syndrome. The World Health Organization (WHO) estimates the total number of TB cases globally at 10 million, with 1.2 million annual deaths (1). The current rate of global TB decrease (1 to 2%) will not be sufficient to achieve the WHO’s End TB goal of eliminating TB by 2035 (2). Moreover, the emergence of multidrug-resistant (MDR) and extensively drug-resistant (XDR) TB has exacerbated the scale of this challenge (1). Consequently, there is an urgent need for new anti-TB drugs and combination regimens to reduce treatment duration and combat drug-resistant disease.

Pulmonary alveolar macrophages and the epithelial lining fluid (ELF) have been identified as important sites of infection for numerous diseases, including TB, which is caused by Mycobacterium tuberculosis (M. tuberculosis). Following cellular uptake, M. tuberculosis can be sequestered into subcellular compartments, including phagolysosomes, lysosome, and cytosol (3, 4), which further alter M. tuberculosis replication rates and susceptibility to antibiotic treatment (5, 6). The sterilization of the intracellular environment should therefore be the goal of drug discovery programs (7–10), particularly given the potential that novel drugs targeting specific intracellular bacilli may hold for shortening treatment (11–14).

Tamm-Horsfall protein 1 (THP-1) is a spontaneously immortalized human monocyte-like cell line (15) which has been widely applied in investigations of intracellular M. tuberculosis infection (16, 17). An advantage of this model is that macrophage-like cell lines display representative features of *in vitro*-differentiated monocyte-derived macrophages (18) while obviating the potential confounder of donor variability in macrophage function. In addition, THP-1 cells can be grown reproducibly, can be studied at different stages (e.g., resting versus activated), are easily infected, and closely model alveolar macrophages for M. tuberculosis-induced apoptosis (19).

The epithelial lining fluid has recently been identified as an important site to measure drug concentrations owing to its role as potential bacterial reservoir (20, 21). Concentrations of antibiotic drugs in lung fluids, including sputum, respiratory tract secretions, bronchial mucosa, epithelial lining fluid, and bronchoalveolar lavage (BAL) fluid, have been measured using a number of different methods (22, 23). Tissue samples are often homogenized, and while they provide a good average concentration of overall drug penetration into organs, information on specific compartmental penetration is lost owing to the processing of organ samples, sometimes leading to poor prediction of target-site drug concentration (24). A significant impediment to TB treatment is caused by the complexity of TB pathology, with the disease progressing from the crucial incipient macrophage infection through to lesion development (25). Drug concentrations in epithelial lining fluid and macrophages provide the best predictive values of target-site concentrations because of their proximity to the initial target site of mycobacterial infection. However, obtaining these samples is often difficult and uncomfortable for patients, involving the insertion of a bronchoscope and washing of the lung environment (26). Nevertheless, the BAL procedure allows for sampling of the epithelial lining fluid and the associated cellular fraction within this fluid, consisting predominantly of monocytic/macrophage cells (27) and providing an ideal *in vivo* comparator for our *in vitro* intracellular model.

**FIG 1:**
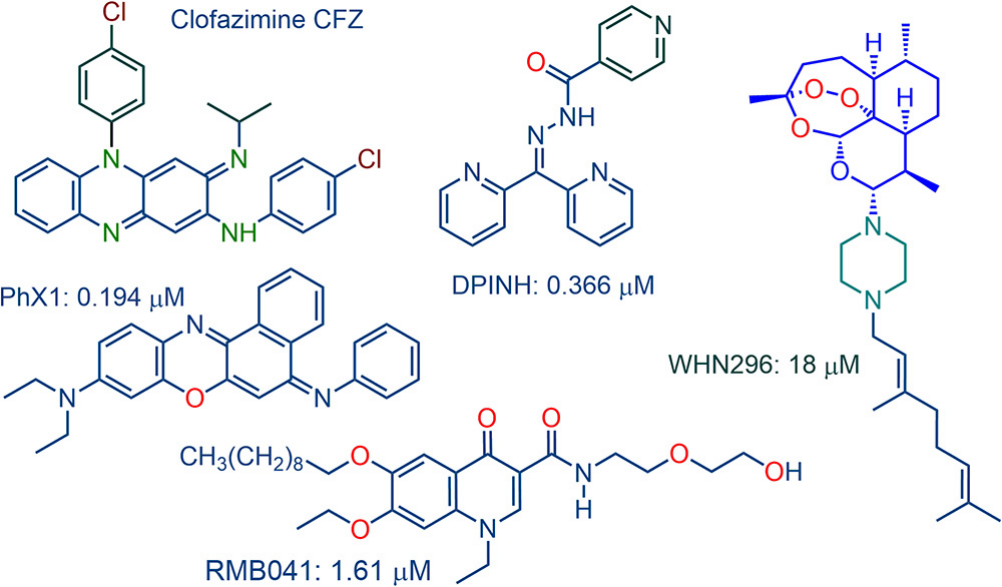
Compounds used in this study with corresponding *in vitro* activities (MIC_90_) against M. tuberculosis H37Rv.

In this study, we evaluated the intracellular accumulations and efficacies as well as potential synergizing activities of the phenoxazine derivative PhX1 (M. tuberculosis MIC_90_ 0.19 μM [28]), the prenylated artemisinin derivative WHN296 (MIC_90_ 18 μM [29]), the isoniazid-based semicarbazone DPINH (MIC_90_ 0.36 μM [30]), and the decoquinate derivative RMB041 (MIC_90_ 1.61 μM [31]), as presented in Figure 1. Clofazimine, which structurally resembles the phenoxazine PhX1, was included as a comparator drug. Both compounds are lipophilic and weakly basic. As such, these drugs, with their lysosomal trapping capacity, tend to accumulate in acidic organelle compartments via pH-dependent ion trapping mechanisms (32). Importantly, clofazimine is equipotent *in vitro* against both MDR and drug-sensitive M. tuberculosis, due to its activity on the M. tuberculosis type II NADH:quinone oxidoreductase (M. tuberculosis NDH2), where it induces the formation of reactive oxygen species via redox cycling that is associated with the increased consumption of NADH (33, 34). Clofazimine-like compounds are ideal for inclusion in this regimen, as both MDR and drug-sensitive TB patients could be treated using similar treatment regimens. In the context of the amino-artemisinin derivative WHN296, it is noted that an artemisinin derivative specially tailored to be taken up via an active transporter into M. tuberculosis (35) and a lipophilic steroidal tetroxane analogue (36) have previously been shown to be active against M. tuberculosis *in vitro*. More recently, amino-artemisinins and tetraoxanes bearing diamine linkers attached to a pyrimidine showed substantial growth suppression of M. tuberculosis cultures *in vitro* (37).

The examination of the anti-M. tuberculosis-active phenoxazines, semicarbazones, decoquinate derivatives, and amino-artemisinin derivatives in comparison with known TB drugs is therefore warranted in the special context of intracellular accumulation, as is now described.

## RESULTS

### PhX1 accumulation in murine bronchoalveolar lavage fluid.

**LC-MS/MS assay performance.** A liquid chromatography tandem mass spectrometry (LC-MS/MS) assay was used for the analysis of PhX1 in infected THP-1 samples with calibration standard and quality control (QC) accuracy (%Nom) between 89.5 ± 7.4 and 110.4 ± 2.2%. This indicated that the calibration curve values for the murine BAL fluid, plasma, and alveolar macrophage samples were well within the acceptable 20% deviation used in this study for both calibration curve and QC values and that the LC-MS/MS assay performed well in the analysis of these murine samples.

**FIG 2:**
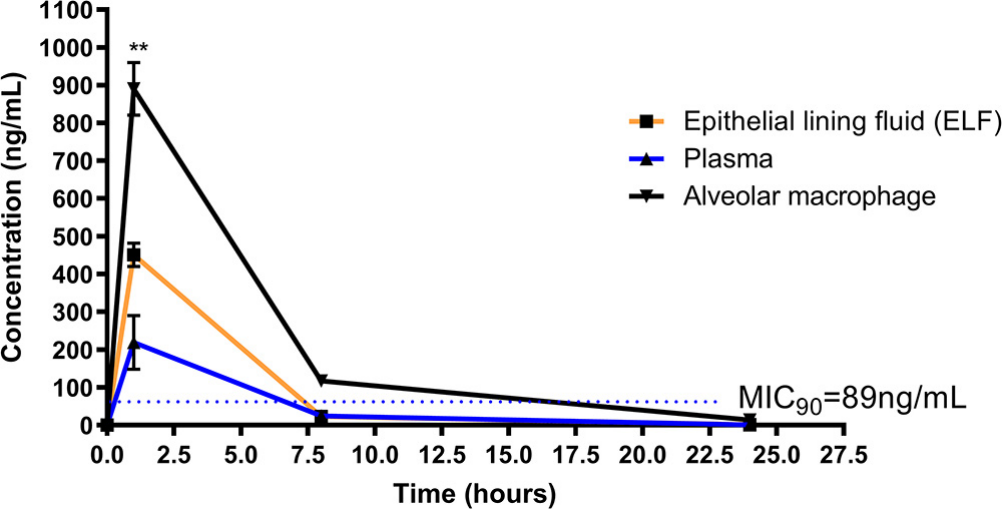
PhX1 concentrations in plasma, epithelial lining fluid (ELF), and alveolar macrophages. Drug concentrations were measured by mass spectrometry over 24 h. Alveolar macrophage PhX1 levels were significantly higher at the 1-h time point compared to plasma levels (*n *= 3 per time point). Compound levels were compared using an analysis of variance (ANOVA) with Dunnett’s *post hoc* test; **, *P* < 0.005.

**PhX1 concentration in lung-associated fluids.** To investigate whether PhX1 accumulated within different lung environments, a BAL procedure was conducted on mice treated with PhX1, with parallel plasma sampling conducted at corresponding time points, followed by LC-MS/MS analysis. This allowed for the determination of compound concentrations in the epithelial lining fluid, alveolar macrophages, and plasma (Fig. 2).

Samples were collected over a 24-h period, and the analysis indicated that PhX1 accumulated to a significantly higher concentration (Fig. 2) in the alveolar macrophages than in the plasma and epithelial lining fluid. PhX1 plasma concentrations in this study were greater than the M. tuberculosis MIC_90_ value for this compound for approximately 6 h, achieving a maximum concentration (*C*_max_) of 200 ± 75 ng/ml. Drug exposure in plasma was calculated using the area under the concentration-time curve (AUC), providing a value of 1,172 ng/ml/h. The concentration of PhX1 in the epithelial lining fluid was significantly higher than that in the plasma, with a time above MIC_90_ of 8 h and a *C*_max_ of 452 ± 22 ng/ml. The epithelial lining fluid AUC was significantly higher than that of plasma at 2,086 ng/ml/h or nearly 2-fold greater than the concentration achieved in plasma.

This disparity between the epithelial lining fluid and plasma is further highlighted by the lung fluid cell concentrations. The predominant cell type within the epithelial lining fluid is pulmonary alveolar macrophages (83%) (23), in which PhX1 accumulates to significant levels (Fig. S1). The concentrations of compound measured in the epithelial lining fluid cells were significantly higher than those recorded in plasma and epithelial lining fluid, achieving a *C*_max_ of 895 ± 23 ng/ml with a calculated AUC of 5,018 ng/ml/h, approximately 4-fold higher than that in plasma samples and 2-fold higher than that in the epithelial lining fluid.

### Distinctive accumulation ratios reported in uninfected THP-1 cells over a 96-h period.

**LC-MS/MS assay performance.** A quadratic regression equation, plotting peak area ratio against concentration, was fitted to the calibration curves. The curves were weighted by 1/concentration (1/*x*). The accuracy (%Nom) for all calibration standards and QC samples was between 91.2 ± 2.5% and 109.1 ± 4.9% in this study. This indicated that calibration curve and QC values were well within the acceptable 20% deviation used in this study for the THP-1 LC-MS/MS cell samples.

**FIG 3:**
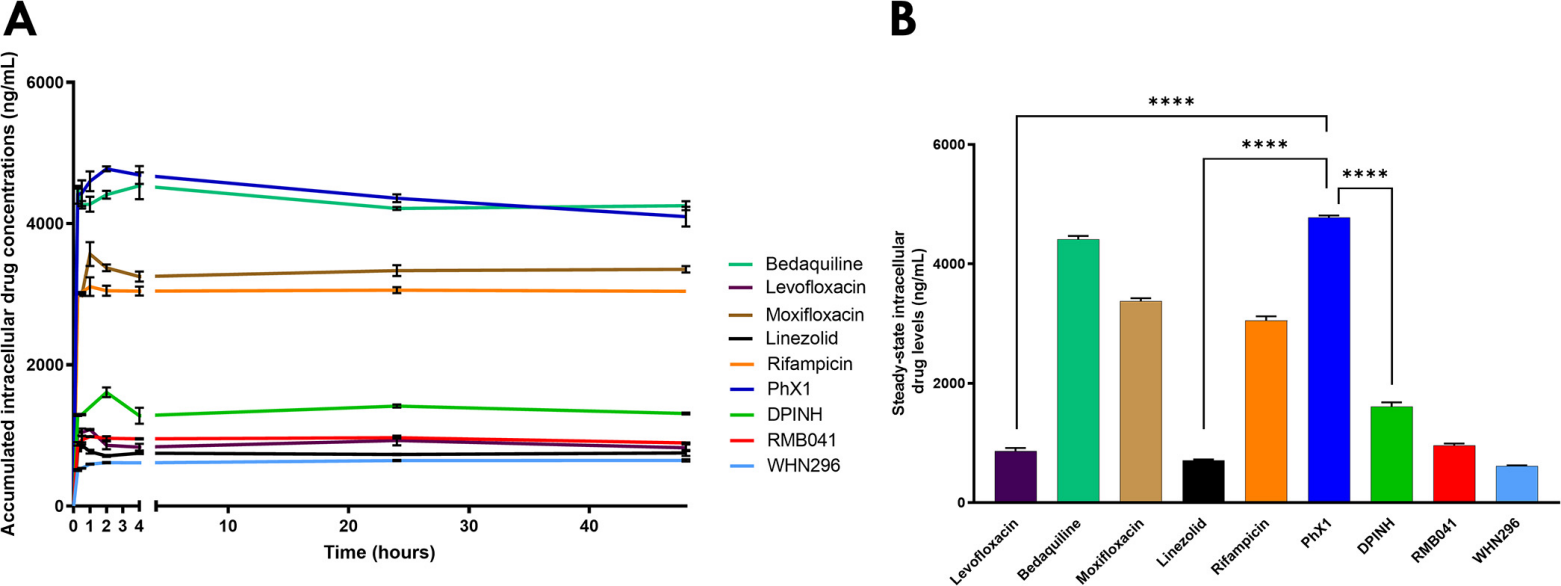
(A) Drug accumulation (intracellular concentrations) in uninfected THP-1 cells following the addition of 5 μg/ml of each compound. Intracellular compound levels were measured over 48 h with compounds displaying variable levels of accumulation. (B) Assumed steady-state concentrations for each compound were used to assess statistical significance of the observed differences (*n* = 3 per experiment; ANOVA with Dunnett’s *post hoc* test comparing steady-state concentrations to PhX1 was used; ****, *P* < 0.0001).

Further investigation into the macrophage-accumulation of PhX1 was conducted using an *in vitro* THP-1 cell model and treated with PhX1 and other experimental and approved TB drugs over a period of 48 h (Fig. 3A). Cell number and viability were unaffected by the addition of drug for the period of 48 h, with cell numbers maintained at values greater than 4 × 10^5^ cells/well for all samples. PhX1 displayed the greatest level of intracellular accumulation (4,750 ± 127.2 ng/ml), with bedaquiline (4,410 ± 190.9 ng/ml), moxifloxacin (3,374 ± 48.7 ng/ml), and rifampin (3,050 ± 62.9 ng/ml) displaying relatively high intracellular compound levels (Fig. 3B). Lower intracellular concentrations were noted for WHN296 and RMB041 and for the known drugs linezolid and levofloxacin (Table 1). Cell viability remained consistent over the course of the experiment, with viability greater than 85% for all samples (Table 1). Statistical analysis revealed that PhX1 accumulated to significantly higher levels than the experimental compounds and several of the clinical drugs included in this study.

**TABLE 1:** Intracellular accumulation and viability in THP-1 cells treated with 5 μg/ml of each compound

Compound	Equilibrium level reached (ng/ml)	Avg cell viability (%)
Levofloxacin	860 ± 56.5	95 ± 4.2
Bedaquiline	4,410 ± 190.9	93 ± 4.9
Moxifloxacin	3,374 ± 48.7	87 ± 2.7
Linezolid	770 ± 14.1	92 ± 3.1
Rifampin	3,050 ± 62.9	89 ± 2.4
PhX1	4,750 ± 127.2	92 ± 5.8
DPINH	1,280 ± 113.1	91 ± 6.2
RMB041	953.5 ± 28.2	97 ± 2.5
WHN296	613.5 ± 19.2	98 ± 1.2

### Phenoxazine accumulation in Mycobacterium smegmatis-infected THP-1 cells.

To investigate whether intracellular accumulation of PhX1 was related to compound structure, several phenoxazine derivatives were assessed for their ability to accumulate in M. smegmatis-infected THP-1 cells. M. smegmatis was used as a bench-safe alternative to M. tuberculosis H37Rv, allowing the development of an assay measuring intracellular accumulation and other readouts in an infection-based system. Multiple samples were taken to assess the time-dependent nature of this accumulation, but no change was seen following 1 h of compound incubation.

**FIG 4:**
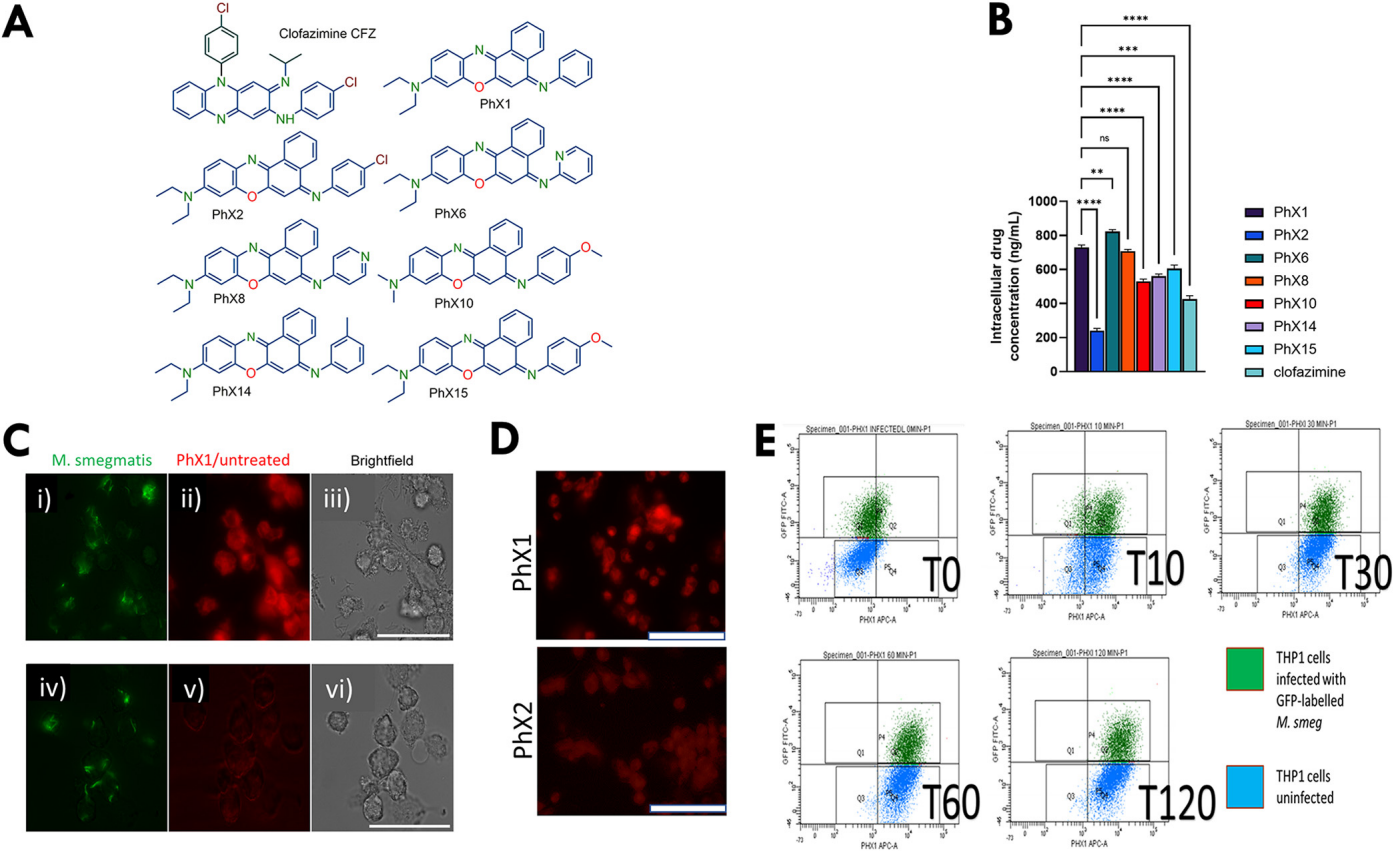
Drug accumulation (intracellular concentrations) in M. smegmatis (*Msm*)-infected THP-1 cells following the addition of 1 μg/ml of each compound. Structures of the PhX compounds are given in panel A. Compound accumulation was assessed using (B) mass spectrometry, (C and D) fluorescence imaging, and (E) flow cytometry. (B) Compound accumulation levels were measured at 2 h postdrug addition (*n* = 3 independent experiments; compound levels were compared to PhX1 using a Dunnett’s test; **, *P* < 0.005 and ****, *P* < 0.0001). (C) THP-1 cells were infected with GFP-expressing M. smegmatis (i and iv) and then treated with 1 μg/ml of PhX1 (ii) or left untreated (v), with cell viability confirmed using brightfield microscopy (iii and vi). Scale bar 100 μm. (D) THP-1 cells treated with PhX1 and PhX2 for 2 h (scale bar 100 μm). (E) Flow cytometry of M. smegmatis (GFP-labeled)-infected THP-1 cells treated with PhX1 over a period of 2 h, with red fluorescence measured in the APC channel (*x* axis) and GFP measured in the FITC channel (*y* axis). M. tuberculosis efficacies and ADME-PK of phenoxazines PhX1, -2, -6, -8, -10, and -14 reported in Tanner et al. (118).

PhX1 accumulated to levels significantly higher than those of the majority of the other phenoxazines (Fig. 4A and B), with PhX6 being the only compound accumulating to higher levels within this model.

To visualize compound accumulation, we conducted fluorescence imaging on M. smegmatis-infected THP1 cells treated with PhX1 or phosphate-buffered saline (PBS)/dimethyl sulfoxide (DMSO) (untreated) as shown in Figure 4C and D. Treatment with PhX1 resulted in intense red fluorescence, with comparatively less fluorescence seen in the untreated sample, indicative of accumulation within these cells (Fig. 4C and D). However, no significant change in M. smegmatis*-*associated green fluorescence could be seen following PhX1 treatment, possibly indicating a lack of efficacy against M. smegmatis as reported for other structurally related compounds (38). This accumulation was confirmed using flow cytometry, with the uninfected and infected THP-1 cells accumulating PhX1 as indicated by the population movement across the allophycocyanin (APC) channel (Fig. 4E).

### M. tuberculosis H37Rv-infected macrophage model.

The approach used in this study focused primarily on a standardized 20× MIC compound dose comparison to incorporate the assay into our screening cascade. This translated into concentrations similar to those achieved in the plasma and epithelial lining fluid for a number of the compounds.

**FIG 5:**
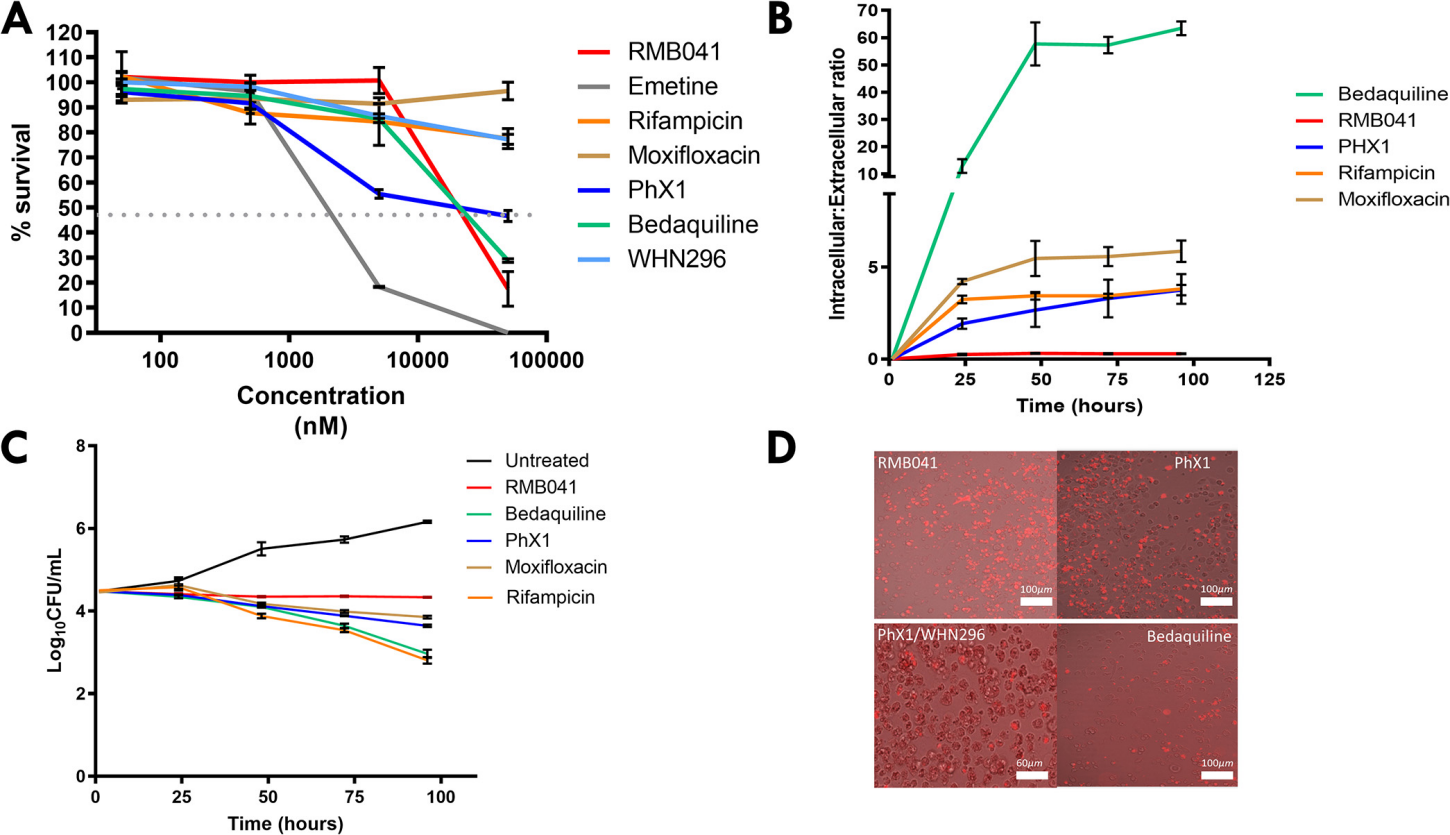
Compound toxicities against THP-1 cells (A), intracellular compound accumulations (B), and intracellular compound efficacies (C and D) against M. tuberculosis. Compound toxicity against THP-1 cells (A) was assessed using an MTT assay (data presented as mean survival ± SD; *n* = 3 per compound). Compound intracellular accumulation (B) and intracellular efficacy (C and D) were assessed in an M. tuberculosis*-*infected THP-1 cell model (*n* = 3 independent experiments; data presented as mean ± SD). Compound efficacy (D) was confirmed visually using fluorescence microscopy, with mCherry-labeled M. tuberculosis (MOI: 5; scale bar 100 μm).

**THP-1 viability.** Compounds were tested at 20× MIC for each compound in the infected THP-1 model, which for some compounds was a relatively high concentration potentially inducing cytotoxicity with concentrations of bedaquiline (1,390 ng/ml), PhX1 (1,780 ng/ml), rifampin (180 ng/ml), moxifloxacin (1,050 ng/ml), RMB041 (15,456 ng/ml), WHN296 (201,600 ng/ml), and clofazimine (2,367 ng/ml) used in each of the corresponding intracellular accumulation assays. Therefore, cytotoxicity of these drugs was subsequently tested in THP-1 cells (5 × 10^5^ cells/well) via an MTT [3-(4,5-dimethyl-2-thiazolyl)-2,5-diphenyl-2H-tetrazolium bromide] assay for a period of 7 days (Fig. 5A). Values of the 50% inhibitory concentration (IC_50_) for the cytotoxicities of the drugs in this assay ranged from 10 μM to greater than 50 μM (Fig. 5A).

**LC-MS/MS assay performance.** The accuracy (%Nom) of the calibration and QC samples ranged between 87.2 ± 3.1 and 108.7 ± 5.7. This indicated that the M. tuberculosis*-*infected THP-1 cell calibration curves were within the acceptable 20% deviation for both the calibration curves and QC values and that the infected THP-1 cell assay could be used to accurately assess M. tuberculosis-infected samples.

**Intracellular drug accumulation in** **M. tuberculosis-infected THP-1 cells drives efficacy.** An intracellular accumulation ratio, defined as the intracellular compound concentration divided by the extracellular concentration, was used in this study to allow comparison between compounds (Fig. 5B). This required the measurement of both intracellular and extracellular concentrations of drug as shown in a number of publications (39–47). RMB041 showed the lowest levels of intracellular accumulation at 0.25, PhX1 displayed accumulation levels of around 2.5 to 3 (similar to rifampin), and moxifloxacin and bedaquiline showed the greatest accumulation in the infected THP-1 cells (5.4 and 5.5, respectively). The known drugs rifampin (41, 43), moxifloxacin (42, 43, 48), and bedaquiline (49, 50) have been shown to achieve similar intracellular accumulation ratios in other studies, validating these results. Additional efficacy measurements were obtained by plating for survival of M. tuberculosis H37Rv (Fig. 5C and D). The untreated control displayed an increased bacterial count of 1.6 log_10_CFU/ml compared to the starting bacterial count. In order of increasing efficacy, RMB041 displayed a decreased bacterial count of 0.1 log_10_CFU/ml, moxifloxacin (−0.5 log_10_CFU/ml), PhX1 (−0.8 log_10_CFU/ml), bedaquiline (−1.5 log_10_CFU/ml), and rifampin (−1.5 log_10_CFU/ml).

### Synergistic interaction in the M. tuberculosis-infected THP-1 model.

**FIG 6:**
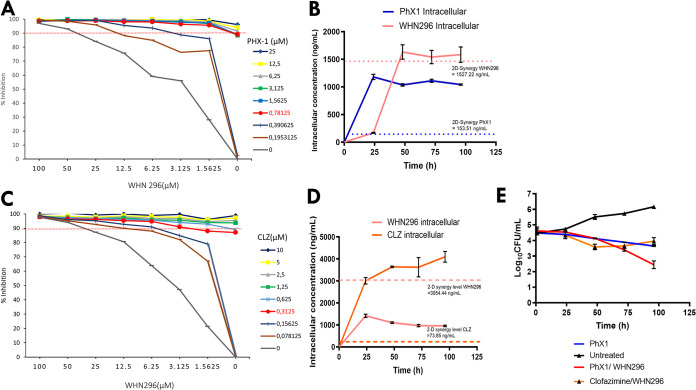
*In vitro* synergy, *in vitro* THP-1 accumulation. and intracellular efficacy. Compound synergy was assessed for two different drug combinations, PhX1/WHN296 and clofazimine (CLZ)/WHN296 (A and C), using a standard two-dimensional synergy assay. Compound intracellular accumulation (B and D) and intracellular efficacy (E) were assessed in an M. tuberculosis*-*infected THP-1 cell model (*n* = 3 independent experiments; data presented as mean ± SD).

A standard two-dimensional (2D) checkerboard/synergy assay was performed to investigate potential synergism between the compounds (Fig. 6A and C). Drug interactions were assessed by checkerboard dilution in a 96-well format with slight modification of the method described by Omollo et al. (51). PhX1 was serially diluted in RPMI medium along the *x* axis (columns 3 to 11), with synergistic concentrations ranging from 25 to 0.19 μM, while WHN296 was serially diluted across the *y* axis (rows B to H), with synergistic concentrations ranging from 100 to 0.15 μM.

The PhX1/WHN296 combination displayed a fractional inhibitory concentration index (FICI) of 0.21 (Fig. 6A), which is defined as synergistic. The minimum values required to produce synergy were also noted for each compound, including PhX1 (153.1 ng/ml) and WHN296 (1,527.2 ng/ml). The results compared well with those obtained from the clofazimine/WHN296 combination, which also produced an FICI of 0.32 (Fig. 6C).

It was necessary to confirm if synergism between PhX1 and WHN296 was conserved in M. tuberculosis-infected THP-1 cells. To compare these results to the single-dose efficacy and combinations obtained using the individual drugs, each compound was dosed at 20× MIC_90_ (Fig. 6B and D). The intracellular accumulation of the combination of PhX1 and WHN296 in the M. tuberculosis-infected THP-1 cell model was evaluated using quantitative LC-MS/MS at time points of 24, 48, 72, and 96 h (*n* = 3 for each time point). In this experiment, intracellular drug concentrations are reported in ng/ml to allow comparisons to be made with the minimum compound levels required to achieve synergy. For the PhX1/WHN296 combination, both compounds reached their respective intracellular synergy levels after approximately 48 h and maintained these levels until the 96-h time point.

Next, it was important to establish whether both drugs in the PhX1/WHN296 combination reaching these intracellular 2D levels resulted in a corresponding increase in efficacy compared to that of PhX1 dosed alone. In this experiment, CFU counts (Fig. 6E) were determined at time points of 24, 48, 72, and 96 h (in triplicate). PhX1 alone and the PhX1/WHN296 combination showed similar CFU counts until the 48-h time point. Thereafter, the PhX1/WHN296 combination showed significantly increased efficacy at the remaining 72- and 96-h time points.

## DISCUSSION

The success of M. tuberculosis as an infectious agent derives from its ability to persist within the human host (52). One of the most distinct features of M. tuberculosis is its ability to infect the macrophage, recently summarized by VanderVen et al. as the “minimal unit of infection” (53). This description encompasses both the innate immune response toward M. tuberculosis and the pathogenicity of M. tuberculosis. In addition, Dartois et al. eloquently outlined the need to understand how individual compounds distribute into the different host cell environments (8). It is generally acknowledged that in order to predict how a drug will respond in the human body, the pharmacokinetic/pharmacodynamic relationship in various tissues and cells must be understood (10, 54, 55). Given the propensity of M. tuberculosis to occupy different microenvironments of the body, it is essential that efforts are focused on developing drugs that are active in all environments.

This was addressed by collecting BAL fluid and plasma from uninfected C57BL/6 mice dosed with compounds over 24 h. Within the context of a therapeutic window, the importance of determining target-site concentrations becomes clearer (55). The evaluation of plasma concentrations in this study would enable us to establish if concentrations are maintained above the MIC for the compounds over a maximum of 7 h. However, for concentrations in the epithelial lining fluid cells, the time during which compound concentrations are above the MIC_90_ would be closer to 15 h.

The ratio between epithelial lining fluid and plasma concentrations is a commonly used predictor of penetration into the target site and is usually presented as a log ratio, with a higher log ratio indicative of higher concentrations in epithelial lining fluid compared to those in the plasma (56, 57). This log ratio for PhX1 (0.65) was higher than that for ciprofloxacin (58–60) at a ratio of −0.2 and similar to isoniazid (61) at a ratio of 1, linezolid (62–64) at 0.7, and rifampin (65, 66) at 0.7, while ethionamide (67) at 2.8, moxifloxacin (68) at 1.5, and pyrazinamide (69) at 3.2 showed significantly higher ratios, indicative of their superior intrapulmonary penetration. However, recent literature points to several flaws associated with determining an epithelial lining fluid/plasma concentration using single-point values such as the *C*_max_ (57, 70, 71). The systemic hysteresis associated with both epithelial lining fluid and plasma compound concentrations requires a more stable pharmacokinetic (PK) parameter to be determined, such as an AUC measurement. In this study, the ELF_AUC_/plasma_AUC_ for PhX1 provided a value of 1.7, which still indicated moderate to high penetration and subsequently high level of epithelial lining fluid exposure compared to those of the clinically used compounds.

Given the cellular accumulation of PhX1 *in vivo*, further investigations were conducted using an *in vitro* THP-1 cell model and compared to intracellular accumulation of known drugs and new compounds, with PhX1 accumulating to greater levels than the majority of other compounds. To determine whether this was related to a structurally distinct feature of PhX1, several phenoxazine derivatives were tested in an M. smegmatis-infected THP-1 cell model. PhX1 displayed higher intracellular accumulation than the majority of the other phenoxazines. Given the 2-fold increase in PhX1 efficacy against *in vitro* M. tuberculosis over PhX6, it was decided that PhX1 would be followed in subsequent experiments. PhX1 LC-MS/MS results were further complemented using flow cytometry, which demonstrated the clear accumulation of PhX1 over time in the APC channel. This supported the accumulation seen in the fluorescence microscopy experiments using untreated and PhX1-treated M. smegmatis*-*infected cells. Drug accumulation over time was recorded using flow cytometry and LC-MS/MS analyses, with a steady state being achieved for both after 2 h.

Evaluation of patterns of drug accumulation within immune cells involved in granuloma formation, including macrophages, indicates that compound accumulation is not straightforward (8, 25, 72). Four distinct patterns of drug penetration have been identified, including (i) rapid and homogeneous distribution with the absence of accumulation appearing over time (isoniazid/linezolid), which may explain the predominant killing of extracellular bacteria by isoniazid, (ii) rapid and heterogeneous distribution with a high degree of accumulation in the cellular rim rather than the caseum (fluoroquinolones), (iii) slower distribution with gradual accumulation of drug over time (rifampin), and (iv) rapid distribution with significant accumulation in the cellular layers and poor diffusion into the caseum (clofazimine and bedaquiline). Rapid homogeneous distribution followed by a lack of accumulation is associated with linezolid, with an equilibrium level being reached within 15 min. It also has been shown elsewhere that linezolid displays intracellular concentrations lower than those of the majority of other compounds (73, 74). The fluoroquinolones accumulate within a variety of cells, including THP-1 cells (75–77). Here, the uptake of the fluoroquinolones moxifloxacin and levofloxacin conformed to the rapid and heterogenous pattern of accumulation, with moxifloxacin accumulating to a slightly higher degree than levofloxacin. Others have compared the degree of accumulation of several fluoroquinolones at steady state (78), which was reached at approximately the same time as in our study (30 min). The frontline anti-TB drug rifampin accumulated exceedingly slowly but reached significant levels in the cellular regions of the granuloma. Rifampin attained high concentrations within THP-1 cells. Bedaquiline has been shown to attain high levels of accumulation within the cellular rim of granulomas and in THP-1 cells (79). Here, it displayed the same degree of accumulation, with equilibrium levels greater than 4 μg/ml being reached within 30 min. The compounds PhX1, DPINH, RMB041, and WHN296 (Fig. 1) displayed various degrees of accumulation. PhX1 showed relatively high levels of accumulation, comparable with the structurally similar clofazimine, which accumulates to high levels within various models (8, 72, 80). The accumulation levels measured for PhX1 were also similar to those of bedaquiline.

Comparatively lower accumulation was observed for DPINH. As noted above, this could be because of the potential of the compound to undergo metabolism to isoniazid, a compound with relatively poor cellular accumulation properties (7, 49). RMB041 and WHN296 accumulated to relatively lower levels within uninfected THP-1 cells, achieving levels similar to those of linezolid and levofloxacin. These differential levels of accumulation were encouraging and allowed us to move forward with the implementation of further perturbations to the model.

M. tuberculosis is a facultative intracellular organism that is able to infect and grow in the macrophage environment (53, 81, 82). The infected THP-1 model is not unique and has been employed using M. smegmatis, Pseudomonas aeruginosa, and various other organisms, including M. tuberculosis (16, 19, 83–85). However, very few studies have been conducted using M. tuberculosis H37Rv as the infectious agent while examining compound accumulation and intracellular efficacy within the same experimental system. The infected macrophage assay was developed based on the uninfected assay used in this study and according to methods described in several publications (8, 43, 49, 84, 86–90). The approach utilized a standardized 20× MIC compound dose comparison to incorporate the assay into our screening cascade. These concentrations presented clinically relevant BAL fluid and plasma concentrations for the majority of the compounds. Rifampin achieves maximum concentrations of approximately 2 μg/ml in the epithelial lining fluid of patients, a dose similar to that used in this study (26, 91). The concentration of moxifloxacin at 5 μg/ml in plasma at the 24-h time point following a once-off 400 mg oral dose in healthy volunteers also compares well with the amount used in our study. However, the *C*_max_ of moxifloxacin achieved in epithelial lining fluid was approximately 10 μg/ml (68, 92, 93), almost double the concentration used in our *in vitro* model. The plasma *C*_max_ achieved for moxifloxacin is approximately 4 μg/ml, a value that more closely resembles the value used in this study (94–96). Bedaquiline, following treatment with a dose of 100 mg, displays plasma concentrations of approximately 0.8 μg/ml, very similar to the 0.6 μg/ml used in this study (97, 98). The PhX1 concentration used during this study was very similar to the maximum concentrations observed in both epithelial lining fluid (0.6 μg/ml) and plasma (1.9 μg/ml) obtained in the murine experiments. The similarities of the starting doses of all the compounds to their *in vivo* concentrations provide a solid grounding for more clinically relevant conclusions to be drawn from these experiments.

Despite moderate to high compound accumulation seen for PhX1 and other compounds, this does not necessarily translate into similar levels of intracellular efficacy (99). This is due to complexities that are increased by cellular involvement, such as drug binding, metabolism, sequestration into organelles, and pH changes. The most notable example of the disconnect between intracellular efficacy and accumulation is reported for moxifloxacin, a drug that shows relatively high accumulation within cells but possesses moderate intracellular activity (86, 100). This is again demonstrated in the current study with moxifloxacin displaying moderate efficacy relative to its accumulation, with approximately 0.5 log_10_CFU/ml difference from the starting log_10_CFU/ml in the 96-h assay; this is similar to the case of PhX1, which displayed a 0.8 log_10_CFU/ml decrease. This contrasts with bedaquiline, which showed significant accumulation in infected THP-1 cells and subsequently a log_10_CFU/ml difference of 1.5. Although rifampin did not show the same degree of accumulation, the compound still achieved a bacterial decrease of approximately 1.5 log_10_CFU/ml, highlighting the need to capture both efficacy and accumulation measurements in the same experiments. The PhX1/WHN296 combination showed CFU counts similar to those of the PhX1 alone up until the 48-h time point. Thereafter, the PhX1/WHN296 combination showed significantly more efficacy than the comparator compounds at the remaining 72- and 96-h time points, decreasing bacterial counts by 2 log_10_CFU/ml. This was possibly due to the synergy levels being reached by each compound at the 48-h time point, translating into an increase in efficacy at the same time point. Conversely, synergy levels of the clofazimine/WHN296 combination were reached only by clofazimine and not by WHN296 following the addition of these compounds, resulting in an increase in CFU counts until after the 48-h time point. The redox-active phenothiazine methylene blue, which is structurally related to the phenoxazine PhX1, potently synergizes the action of artemisinins against the malaria parasite through enhanced production of reactive oxygen species (ROS), as described in detail elsewhere (101). Given that M. tuberculosis has a well-defined oxidative stress response (102–104), it was expected that the redox-active phenoxazine PhX1 or the structurally related clofazimine (34), in combination with the amino-artemisinin WHN296, would promote the production of ROS, thus explaining the synergism seen against M. tuberculosis.

This study supports the use of the combination of PhX1/WHN296, or indeed more lipophilic artemisinin derivatives (29), in further studies given the large degree of both *in vitro* and *in vivo* accumulation and efficacy in the *in vitro* intracellular assays. The model, combining two-dimensional synergy, mass spectrometry, and efficacy evaluation, provides a novel approach to understanding synergistic efficacy and accumulation within the infected macrophage. These combinations may synergistically potentiate intracellular activities of the components offering attractive and more flexible options for combatting clinical TB disease. In addition, it is clear that use of drug combinations capable of enhancing oxidative stress (104) has considerable potential and requires further evaluation according to the methods disclosed here.

## MATERIALS AND METHODS

### Ethics.

All animal studies were conducted with approval from the Animal Ethics Committee of the University of Cape Town (013/032). The experiments were conducted in accordance with the National Code for Animal Use in South Africa (105).

### Materials and chemicals.

Potassium dihydrogen phosphate and dipotassium hydrogen phosphate were obtained from Merck (Kenilworth, NJ, USA). Liquid chromatography-mass spectrometry (LC-MS)-grade acetonitrile (acetonitrile) was purchased from Anatech (Johannesburg, South Africa). Bedaquiline, high-performance liquid chromatography (HPLC)-grade dimethyl sulfoxide (DMSO), and formic acid were obtained from Sigma-Aldrich (St. Louis, MO, USA). The compounds PhX1, PhX2, PhX6, PhX8, PhX10, PhX14, PhX15, and DPINH were prepared as reported previously (30, 106–110). Compounds were synthesized according to the methodologies reported by Crossley et al. unless otherwise stated (106, 111). RMB041 and WHN296 were synthesized according to previously reported methodologies (29, 112). Water was purified using a Milli-Q purification system (Millipore, Bedford, MA, USA).

### Bronchoalveolar lavage fluid analysis.

**Animals.** Male C57BL/6 mice, 12 to 16 weeks old, weighing approximately 30 g, were acclimatized to their environment 4 days prior to the start of the experiment. Mice were maintained at the animal facility of the University of Cape Town and were fed a standard laboratory diet and water *ad libitum*. Mice were housed in 27 by 21 by 18 cm cages, under controlled environmental conditions (26 ± 1°C with 12-h light/dark cycles).

**Dosing.** Compounds were prepared in theoretical fixed volumes for the average weight. Dose volumes ranged from 200 to 300 μl for oral dose. Animals (*n* = 9) were dosed at 10 mg/kg (based on weight), and three animals were dosed with vehicle control (100% hydroxypropyl methylcellulose [HPMC]) in order to obtain blank bronchoalveolar lavage fluid for calibration curves.

**Bronchoalveolar lavage fluid procedure.** Mice were anesthetized via intraperitoneal (i.p.) injection of ketamine/xylazine (75 to 100 mg/kg plus 10 mg/kg) at predetermined time points (1, 8, and 24 h postdrug administration). Depth of anesthesia was monitored by the absence of the pedal withdrawal reflex. Ten min before the bronchoalveolar lavage fluid procedure, blood samples (each 30 μl) were drawn from the mice by tail tip bleeding and placed in tubes containing EDTA. This sample was immediately spun down (5 min, 10,621 × *g*) to obtain plasma. Mice were prepared for the surgical procedure by shaving and washing of the neck area. Dissection of the neck tissue to expose the trachea was completed to allow a small tracheal incision allowing the passage of a 23-gauge intravenous (i.v.) catheter into the trachea. PBS (100 μl) was introduced into the lungs of the animals via the lavage tube, aspirated four times inside the lung, and removed. Samples were collected on ice, and the total volume collected was recorded. Exsanguination via cardiac puncture was carried out postprocedure.

**Sample processing.** Cell numbers were determined using trypan blue staining and a TC-20 automated cell counter (Bio-Rad, CA, USA). Bronchoalveolar lavage fluid samples were then processed by initially spinning down (5 min, 10,621 × *g*) the samples to pellet any remaining cells, followed by removal of the supernatant.

**ELF volume determination.** Bronchoalveolar lavage fluid supernatant was analyzed using the colorimetric Quantichrom urea assay kit DIUR-500 (BioAssay systems, CA, USA). The optical density (OD) of each sample was read using a Bio-Rad iMark microplate absorbance reader (Bio-Rad, CA, USA). A calibration curve was constructed using these readings, and quantitative estimations of urea concentration in both the plasma and bronchoalveolar lavage fluid were determined using this calibration curve. Epithelial lining fluid volume was then determined using equation 1 below.

Determination of epithelial lining fluid volume in murine bronchoalveolar lavage fluid:

(1) $$V_{\text{ELF}}=V_{\text{BAL}}\times {\left[\mathrm{urea}\right]}_{\text{BAL}}/{\left[\mathrm{urea}\right]}_{\text{plasma}}$$

**Sample processing leading to LC-MS/MS analyses.** Cellular, plasma, and lung fluid samples were processed by the addition of acetonitrile containing 1 μg/ml of internal standard (carbamazepine) to sample, followed by vortexing (1 min) and centrifugation (5 min, 10,621 × *g*). Supernatants were then removed, dried down under nitrogen, and reconstituted for LC-MS/MS analysis using injection solvent.

Standards and QC samples were prepared in each matrix (pooled blank murine matrices) in triplicate and were extracted using the same acetonitrile protein precipitation method as that described above. LC-MS/MS analysis was performed. Briefly, an Agilent 1200 rapid resolution HPLC system comprising a binary pump, a degasser, and an auto-sampler (Agilent, Little Falls, Wilmington, USA) coupled to an AB Sciex 4000 QTrap hybrid triple quadrupole linear ion-trap mass spectrometer (AB Sciex, Framingham, MA, USA) was used for sample analysis. Epithelial lining fluid concentrations were subsequently calculated for each mouse using equation 2 below.

Concentration of drug in epithelial lining fluid (ELF):

(2) $${\left[\text{compound}\right]}_{\text{ELF}}={\left[\text{compound}\right]}_{\text{BAL}}\times V_{\text{BAL}/\text{plasma}}$$

### Uninfected macrophage assay.

**Cell culture and drug addition.** Tamm-Horsfall protein 1 cells (THP-1) were plated in 24-well plates, followed by the addition of 0.1 μM phorbol 12-myristate 13-acetate (PMA). The cells were then supplemented with RPMI medium containing 10% fetal calf serum (FCS). Cells were grown at 37°C in 5% CO_2_. RPMI containing 20× M. tuberculosis MIC_90_ of the drugs (compound-specific) was added in a volume of 500 μl, in triplicate. This was followed by washing of cells using RPMI medium and was repeated thrice. EDTA was used to detach cells from the surface of the plate. Cell numbers were determined using a trypan blue staining method and a TC-20 automated cell counter (Bio-Rad, CA, USA). Cells were then lysed using 100 μl chloroform/methanol (3:1, vol/vol) and 1 μg/ml of compound-specific internal standard (deuterated internal standard for known anti-TB compounds and carbamazepine for novel compounds).

**LC-MS/MS sample preparation and analysis.** Samples were vortexed (1 min) and centrifuged (5 min, 10,621 × *g*). Sample supernatants were subsequently removed, transferred into a 96-well analysis plate, and dried down under nitrogen. Samples were reconstituted in a mixture of 1:1 H_2_O/acetonitrile (containing 0.1% formic acid [FA], vol/vol) and processed using quantitative LC-MS/MS assays developed at the University of Cape Town. Chromatographic separation was performed using an Agilent 1200 rapid resolution HPLC system consisting of a binary pump, a degasser, and an auto-sampler (Agilent, Little Falls, Wilmington, USA) coupled to an AB Sciex 4000 QTrap, hybrid triple quadrupole linear ion-trap mass spectrometer (AB Sciex, Johannesburg, South Africa). Blank cell lysates (lysed using acetonitrile [0.1% Triton X]) were spiked in triplicate with serially diluted compound concentrations to generate the calibration (2 to 5,000 ng/ml) and quality control (6 to 4,000 ng/ml) samples. These samples were extracted using the procedure described above and were used to construct calibration curves to quantitate the amount of analyte in each respective intracellular sample. Extracellular concentrations were determined by spiking serially diluted concentrations of compound into media removed from the untreated cells to generate calibration and quality control samples and used to determine drug concentrations in the extracellular environment. Quantitative estimations of intracellular drug concentrations were measured using LC-MS/MS. Transitions for bedaquiline, moxifloxacin, rifampin, linezolid, and levofloxacin were monitored at 555.2 to 57.8, 402.2 to 384.0, 823.9 to 791.2, 337.9 to 296.3, and 362.4 to 318.1 *m/z*, respectively. Deuterated internal standards were used for each of the known compounds with transitions monitored for bedaquiline-D6, moxifloxacin-D4, rifampin-D3, linezolid-D3, and levofloxacin-D8 at 561.1 to 64.1, 406.3 to 388.2, 826.5 to 794.4, 340.9 to 297.3, and 370.4 to 326.1 *m/z*, respectively. LC-MS transitions for PhX1, PhX2, PhX6, PhX8, PhX10, PhX14, PhX15, RMB041, WHN296, clofazimine, and the internal standard carbamazepine were monitored at 395.2 to 351.2, 428.9 to 370.4, 395.5 to 341.4, 395.5 to 351.6, 396.5 to 352.4, 408.5 to 350.2, 424.5 to 351.9, 505.2 to 418.0, 489.2 to 223.2, 474.4 to 431.3, and 237.1 to 194.1, respectively. Electrospray ionization, tandem mass spectrometry (MS), and liquid chromatography (LC) elution conditions can be found in the supplementary information (Tables S1 to S3).

***In vitro* cytotoxicity in THP-1 cell lines.** THP-1 cells were routinely maintained as adherent monolayers as reported above. The culture medium was changed every 3 days when the cells were subcultured. Mosmann’s MTT [3-(4,5-dimethylthiazol-2-yl)-2,5-diphenyltetrazolium bromide] assay was used, with minor modifications to determine cell viability (113). After the addition of the compounds at a starting concentration of 50 μM (serial diluted to 5 mM), each well received MTT at a concentration of 5 mg/ml in phosphate-buffered saline (PBS), with blank samples receiving only medium and MTT. Each compound concentration tested in this study was completed in triplicate. The plates were incubated for 4 h at 37°C before centrifuging for 10 min at 500 × *g* and removing the supernatant from the wells without disturbing the formazan crystals. DMSO was added to each well, and the plate was shaken for 5 min on vortex to dissolve the crystals. The absorbance of the formazan salt was measured at 540 nm by a Bio-Rad iMark microplate absorbance reader (CA, USA). The following formula (equation 3) was used to calculate the cell viability:

Cell viability in CHO and VERO cell lines:

(3) $$\text{\% }\mathrm{viability}=\frac{{\mathrm{sample}}_{\mathrm{absorbance}}\;-\;{\mathrm{blank}}_{\mathrm{absorbance}}}{{\mathrm{control}}_{\mathrm{absorbance}}\;-\;{\mathrm{blank}}_{\mathrm{absorbance}}}\times \mathrm{100}$$

Nonlinear dose response curves were constructed using GraphPad Prism 4 software and Microsoft Excel.

**Fluorescence microscopy.** THP-1 cells were seeded in a 24-well plate containing a single pretreated (poly-l-lysine) cover slip at 5 × 10^5^ cells/well. After PMA maturation for 24 h, the cells were infected with a log-phase culture of green fluorescent protein (GFP)-expressing M. smegmatis (OD = 0.6) at a multiplicity of infection (MOI) of 5. The infection was allowed to continue for 30 min, after which the wells were washed with fresh RPMI medium. RPMI medium containing 1 μg/ml of each drug was added to each well, in triplicate. The experiment progressed for an additional 2 h before the wells were again washed with RPMI medium. The coverslips were carefully removed from each well and adhered to a microscope slide containing a single drop of paraformaldehyde. Microscope slides were then examined using an Axio Scope A1 microscope and images were captured using a Zeiss 1 MP monochrome camera (Carl Zeiss, Germany). Samples were exposed to the same exposure settings for respective channels for image acquisition. GFP fluorescence was detected using a 488-nm excitation filter and a 510-nm emission filter set. Red fluorescence from the compounds was detected using a 587-nm excitation laser filter and 610-nm long-pass emission filter. Finally, brightfield imaging was used to detect the position of the bacteria within the macrophage cells.

**Flow cytometry.** Flow cytometry was used to isolate M. smegmatis-infected THP-1 cell populations while simultaneously monitoring PhX1 accumulation in the allophycocyanin (APC) channel (red fluorescence, 650-nm excitation, and 660-nm emission wavelength).

THP-1 cells were seeded at a concentration of 5 × 10^5^ cells/well. After PMA maturation for 24 h, the cells were infected with a log-phase culture of Mycobacterium smegmatis mc2155::*gfp* (OD = 0.6), a mycobacterial reporter strain expressing GFP protein (114, 115) at a multiplicity of infection (MOI) of 5. The infection was allowed to continue for 30 min, after which the wells were washed with fresh RPMI medium. RPMI medium containing 1 μg/ml of drug was added to each well. The experiment was allowed to progress for an additional 2 h before the wells were again washed with RPMI medium. Cells were detached using EDTA (5 mM). Samples were left unfixed, as fixation agents may have caused cell lysis and loss of drug. Sorting was completed using the FACS Jazz cell sorter instrument for 10,000 events per samples (BD, NJ, USA). Gating was initially based on forward and side scatter (size of macrophage) to differentiate debris and undifferentiated cells, followed by further gating based on the live/dead stain SYTOX (used according to the manufacturer’s instructions). Samples were analyzed and further sorted based on GFP fluorescence, providing both uninfected and infected populations of cells. Results were analyzed using the FlowJo software package.

### M. tuberculosis-infected macrophage assay.

THP-1 cells were grown in RPMI medium containing 10% FCS at 37°C in 5% CO_2_. Cells were seeded and differentiated according to the above protocol for 24 h using PMA. M. tuberculosis (H37RvMa) cells were grown to mid-log phase in 7H9 medium. THP-1 cells were infected at an MOI of 5. Following 3 h of infection, wells were washed with RPMI medium to remove extracellular bacteria and dead cells. RPMI medium containing a predetermined concentration of compound was added to each well in triplicate. The solution was allowed to equilibrate for 30 min. In addition, three wells were left without drug treatment (following M. tuberculosis infection) and served as untreated controls, while an additional three wells of THP-1 cells were left uninfected and were used as uninfected controls. Thereafter, at each time point, cells were washed with RPMI medium. This was followed by the addition of EDTA (5 mM), after which 5 min was allowed for the cells to detach. Trypan blue-stained cells were counted using the automated cell counter. The remaining supernatant (150 μl) was added directly to an Eppendorf tube containing chloroform/methanol (3:1, vol/vol) and 1 μg/ml of compound-specific internal standard (deuterated internal standard for known anti-TB compounds and carbamazepine for novel compounds). An aliquot of 5 μl of the remaining supernatant was added to 90 μl RPMI medium, and 10 μl of this solution was streaked onto hygromycin-containing 7H10 plates and placed at 37°C, followed by counting 21 days following plating. After the collection of samples at the remaining time points, the samples were surface-decontaminated and stored at −80°C overnight. The samples were thawed and transferred into a 96-well analysis plate and dried down under a gentle stream of NO_2_ gas. The samples were then reconstituted in a mixture of 1:1 acetonitrile/H_2_O (containing 0.1% FA, vol/vol) before analysis via LC-MS/MS. Samples were processed using quantitative LC-MS/MS assays. Chromatographic separation was performed using an Agilent 1200 rapid resolution HPLC system consisting of a binary pump, degasser, and auto-sampler (Agilent, Little Falls, Wilmington, USA) coupled to an AB Sciex 4000 QTrap, hybrid triple quadrupole linear ion-trap mass spectrometer (AB Sciex, Johannesburg, South Africa). Blank infected cell lysates (lysed using chloroform:methanol) were spiked in triplicate and extracted using the same procedure as that described above to generate compound-specific standard and quality control samples. These were then used to construct calibration curves, which were subsequently used to quantitate the amount of analyte in each respective sample. Extracellular concentrations were determined by spiking serially diluted concentrations of compound in triplicate into media removed from untreated THP-1 cells to generate calibration and quality control samples, which were then extracted using the same procedure as that described above and used to quantitate the amount of compound in each extracellular sample.

### Synergy assay.

Drug interactions were assessed by checkerboard dilution in a 96-well format with slight modifications to the method described by Chen et al. (116). The first column contained drug-free control cells and the last column contained control drug giving maximum inhibition. Compound A was pipetted at starting concentrations 100-fold higher than those in the 96-well plate along the *x* axis (column 3 to 11), and compound B was pipetted at starting concentrations 50-fold higher than those in the 96-well plate (from row B to H). Column 1 and column 12 contained maximum inhibitory concentrations of drug (no bacterial growth) and media containing only M. tuberculosis (maximum bacterial growth), respectively. Rows A3 to A11 contained only PhX1, and columns B2 to H2 contained only WHN296, which allowed for the calculation of MIC_90_ values for each compound. M. tuberculosis cultures were overlaid (100 μl) onto the drug containing wells at a concentration of 1 × 10^8^ cells/ml (OD = 0.6). Plates were incubated at 37°C for 8 days. Following the addition of resazurin, the plates were incubated for a further 3 days to allow a color change from blue to pink, associated with mycobacterial growth, and read at 560 nm on a Spectramax multimode plate reader (Molecular Devices). Synergy was determined by the fractional inhibitory concentration index (FICI) in equation 4.

FICI ratio determination:

(4) $$\mathrm{FICI}\;=\;\mathrm{FICA}\;+\;\mathrm{FICB}\;=\;\frac{\mathrm{CAcomb}}{\mathrm{MICAsingle}}\;+\;\frac{\mathrm{CBcomb}}{\mathrm{MICBsingle}}$$

where CAcomb and CBcomb are the concentrations of drugs A and B which are the iso-effective concentrations (combinations of each drug which produced a level greater than their respective MIC_90_).

FICI values for which the FICI was ≤0.5 represented synergy, FICI of >4 represented antagonism, and any value between 0.5 and 4 represented no interaction (117).

## References

[1] WHO. 2020. Global tuberculosis report 2020. World Health Organization, Geneva, Switzerland.

[2] Schito M, Hanna D, Zumla A. 2017. Tuberculosis eradication versus control. Int J Infect Dis 56:10–13. doi:10.1016/j.ijid.2016.11.007. 27872016

[3] Bussi C, Gutierrez MG. 2019. *Mycobacterium tuberculosis* infection of host cells in space and time. FEMS Microbiol Rev 43:341–361. doi:10.1093/femsre/fuz006. 30916769 PMC6606852

[4] Lerner TR, Borel S, Greenwood DJ, Repnik U, Russell MRG, Herbst S, Jones ML, Collinson LM, Griffiths G, Gutierrez MG. 2017. *Mycobacterium tuberculosis* replicates within necrotic human macrophages. J Cell Biol 216:583–594. doi:10.1083/jcb.201603040. 28242744 PMC5350509

[5] Liu Y, Tan S, Huang L, Abramovitch RB, Rohde KH, Zimmerman MD, Chen C, Dartois V, VanderVen BC, Russell DG. 2016. Immune activation of the host cell induces drug tolerance in *Mycobacterium tuberculosis* both in vitro and in vivo. J Exp Med 213:809–825. doi:10.1084/jem.20151248. 27114608 PMC4854729

[6] Podinovskaia M, Lee W, Caldwell S, Russell DG. 2013. Infection of macrophages with *Mycobacterium tuberculosis* induces global modifications to phagosomal function. Cell Microbiol 15:843–859. doi:10.1111/cmi.12092. 23253353 PMC3620910

[7] Hartkoorn RC, Chandler B, Owen A, Ward SA, Bertel Squire S, Back DJ, Khoo SH. 2007. Differential drug susceptibility of intracellular and extracellular tuberculosis, and the impact of P-glycoprotein. Tuberculosis 87:248–255. doi:10.1016/j.tube.2006.12.001. 17258938

[8] Dartois V. 2014. The path of anti-tuberculosis drugs: from blood to lesions to mycobacterial cells. Nat Rev Microbiol 12:159–167. doi:10.1038/nrmicro3200. 24487820 PMC4341982

[9] Zhang Y, Yew WW, Barer MR. 2012. Targeting persisters for tuberculosis control. Antimicrob Agents Chemother 56:2223–2230. doi:10.1128/AAC.06288-11. 22391538 PMC3346619

[10] Evangelopoulos D, da Fonseca JD, Waddell SJ. 2015. Understanding anti-tuberculosis drug efficacy: rethinking bacterial populations and how we model them. Int J Infect Dis 32:76–80. doi:10.1016/j.ijid.2014.11.028. 25809760

[11] Yuan T, Sampson NS. 2018. Hit generation in TB drug discovery: from genome to granuloma. Chem Rev 118:1887–1916. doi:10.1021/acs.chemrev.7b00602. 29384369 PMC5832989

[12] Machado D, Girardini M, Viveiros M, Pieroni M. 2018. Challenging the “drug-likeness” dogma for new drug discovery in tuberculosis. Front Microbiol 9:1367. doi:10.3389/fmicb.2018.01367. 30018597 PMC6037898

[13] Young DB, Perkins MD, Duncan K, Barry CE. 2008. Confronting the scientific obstacles to global control of tuberculosis. J Clin Invest 118:1255–1265. doi:10.1172/JCI34614. 18382738 PMC2276805

[14] Aljayyoussi G, Jenkins VA, Sharma R, Ardrey A, Donnellan S, Ward SA, Biagini GA. 2017. Pharmacokinetic-pharmacodynamic modelling of intracellular *Mycobacterium tuberculosis* growth and kill rates is predictive of clinical treatment duration. Sci Rep 7:502. doi:10.1038/s41598-017-00529-6. 28356552 PMC5428680

[15] Tsuchiya S, Yamabe M, Yamaguchi Y, Kobayashi Y, Konno T, Tada K. 1980. Establishment and characterization of a human acute monocytic leukemia cell line (THP-1). Int J Cancer 26:171–176. doi:10.1002/ijc.2910260208. 6970727

[16] Fontán P, Aris V, Ghanny S, Soteropoulos P, Smith I. 2008. Global transcriptional profile of *Mycobacterium tuberculosis* during THP-1 human macrophage infection. Infect Immun 76:717–725. doi:10.1128/IAI.00974-07. 18070897 PMC2223452

[17] Madhvi A, Mishra H, Leisching GR, Mahlobo PZ, Baker B. 2019. Comparison of human monocyte derived macrophages and THP1-like macrophages as in vitro models for *M. tuberculosis* infection. Comp Immunol Microbiol Infect Dis 67:101355. doi:10.1016/j.cimid.2019.101355. 31586851

[18] Stokes RW, Doxsee D. 1999. The receptor-mediated uptake, survival, replication, and drug sensitivity of *Mycobacterium tuberculosis* within the macrophage-like cell line THP-1: a comparison with human monocyte-derived macrophages. Cell Immunol 197:1–9. doi:10.1006/cimm.1999.1554. 10555990

[19] Riendeau CJ, Kornfeld H. 2003. THP-1 cell apoptosis in response to mycobacterial infection. Infect Immun 71:254–259. doi:10.1128/IAI.71.1.254-259.2003. 12496173 PMC143334

[20] Scordo JM, Olmo-Fontánez AM, Kelley HV, Sidiki S, Arcos J, Akhter A, Wewers MD, Torrelles JB. 2019. The human lung mucosa drives differential *Mycobacterium tuberculosis* infection outcome in the alveolar epithelium. Mucosal Immunol 12:795–804. doi:10.1038/s41385-019-0156-2. 30846830 PMC6462240

[21] Torrelles JB, Schlesinger LS. 2017. Integrating lung physiology, immunology, and tuberculosis. Trends Microbiol 25:688–697. doi:10.1016/j.tim.2017.03.007. 28366292 PMC5522344

[22] Dhanani J, Roberts JA, Chew M, Lipman J, Boots RJ, Paterson DL, Fraser JF. 2010. Antimicrobial chemotherapy and lung microdialysis: a review. Int J Antimicrob Agents 36:491–500. doi:10.1016/j.ijantimicag.2010.08.013. 20952164

[23] Rodvold KA, Gotfried MH, Still JG, Clark K, Fernandes P. 2012. Comparison of plasma, epithelial lining fluid, and alveolar macrophage concentrations of solithromycin (CEM-101) in healthy adult subjects. Antimicrob Agents Chemother 56:5076–5081. doi:10.1128/AAC.00766-12. 22802254 PMC3457395

[24] Mouton JW, Theuretzbacher U, Craig WA, Tulkens PM, Derendorf H, Cars O. 2008. Tissue concentrations: do we ever learn? J Antimicrob Chemother 61:235–237. doi:10.1093/jac/dkm476. 18065413

[25] Strydom N, Gupta SV, Fox WS, Via LE, Bang H, Lee M, Eum S, Shim T, Barry IIC, Zimmerman M, Dartois V, Savic RM. 2019. Tuberculosis drugs’ distribution and emergence of resistance in patient’s lung lesions: a mechanistic model and tool for regimen and dose optimization. PLoS Med 16:e1002773. doi:10.1371/journal.pmed.1002773. 30939136 PMC6445413

[26] Kiem S, Schentag JJ. 2008. Interpretation of antibiotic concentration ratios measured in epithelial lining fluid. Antimicrob Agents Chemother 52:24–36. doi:10.1128/AAC.00133-06. 17846133 PMC2223903

[27] Heron M, Grutters JC, ten Dam-Molenkamp KM, Hijdra D, van Heugten-Roeling A, Claessen AME, Ruven HJT, van den Bosch JMM, van Velzen-Blad H. 2012. Bronchoalveolar lavage cell pattern from healthy human lung. Clin Exp Immunol 167:523–531. doi:10.1111/j.1365-2249.2011.04529.x. 22288596 PMC3374285

[28] Tanner L, Haynes RK, Wiesner JL. 2020. Accumulation of TB-active compounds in murine organs relevant to infection by *Mycobacterium tuberculosis*. Front Pharmacol 11:724. doi:10.3389/fphar.2020.00724. 32508649 PMC7248252

[29] Wong HN, Lewies A, Haigh M, Viljoen JM, Wentzel JF, Haynes RK, Du Plessis LH. 2020. Anti-melanoma activities of artemisone and prenylated amino-artemisinins in combination with known anticancer drugs. Front Pharmacol 11:558894. doi:10.3389/fphar.2020.558894. 33117161 PMC7552967

[30] Becker EM, Lovejoy DB, Greer JM, Watts R, Richardson DR. 2003. Identification of the di-pyridyl ketone isonicotinoyl hydrazone (PKIH) analogues as potent iron chelators and anti-tumour agents. Br J Pharmacol 138:819–830. doi:10.1038/sj.bjp.0705089. 12642383 PMC1573713

[31] Tanner L, Haynes RK, Wiesner L. 2019. An in vitro ADME and in vivo pharmacokinetic study of novel TB-active decoquinate derivatives. Front Pharmacol 10:120. doi:10.3389/fphar.2019.00120. 30833898 PMC6387968

[32] Baik J, Rosania GR. 2012. Macrophages sequester clofazimine in an intracellular liquid crystal-like supramolecular organization. PLoS One 7:e47494. doi:10.1371/journal.pone.0047494. 23071814 PMC3469554

[33] Sellamuthu S, Singh M, Kumar A, Singh SK. 2017. Type-II NADH dehydrogenase (NDH-2): a promising therapeutic target for antitubercular and antibacterial drug discovery. Expert Opin Ther Targets 21:559–570. doi:10.1080/14728222.2017.1327577. 28472892

[34] Yano T, Kassovska-Bratinova S, Shin Teh J, Winkler J, Sullivan K, Isaacs A, Schechter NM, Rubin H. 2011. Reduction of clofazimine by mycobacterial type 2 NADH: quinoneoxidoreductase: a pathway for the generation of bactericidal levels of reactive oxygen species. J Biol Chem 286:10276–10287. doi:10.1074/jbc.M110.200501. 21193400 PMC3060482

[35] Miller MJ, Walz AJ, Zhu H, Wu C, Moraski G, Möllmann U, Tristani EM, Crumbliss AL, Ferdig MT, Checkley L, Edwards RL, Boshoff HI. 2011. Design, synthesis and study of a mycobactin-artemisinin conjugate that has selective and potent activity against tuberculosis and malaria. J Am Chem Soc 133:2076–2079. doi:10.1021/ja109665t. 21275374 PMC3045749

[36] Šolaja BA, Terzić N, Pocsfalvi G, Gerena L, Tinant B, Opsenica D, Milhous WK. 2002. Mixed steroidal 1,2,4,5-tetraoxanes: antimalarial and antimycobacterial activity. J Med Chem 45:3331–3336. doi:10.1021/jm020891g. 12139444

[37] van der Westhuyzen CW, Haynes RK, Panayides J-L, Wiid I, Parkinson CJ. 2020. Anti-mycobacterial peroxides: a new class of agents for development against tuberculosis. Med Chem 16:392–402. doi:10.2174/1573406415666190430143535. 31208310

[38] Mothiba MT, Anderson R, Fourie B, Germishuizen WA, Cholo MC. 2015. Effects of clofazimine on planktonic and biofilm growth of *Mycobacterium tuberculosis* and *Mycobacterium smegmatis*. J Glob Antimicrob Resist 3:13–18. doi:10.1016/j.jgar.2014.12.001. 27873644

[39] Hand WL, Corwin RW, Steinberg TH, Grossman GD. 1984. Uptake of antibiotics by human alveolar macrophages. Am Rev Respir Dis 129:933–937. doi:10.1164/arrd.1984.129.6.933. 6732052

[40] Prideaux B, Dartois V, Staab D, Weiner DM, Goh A, Via LE, Barry CE, Stoeckli M. 2011. High-sensitivity MALDI-MRM-MS imaging of moxifloxacin distribution in tuberculosis-infected rabbit lungs and granulomatous lesions. Anal Chem 83:2112–2118. doi:10.1021/ac1029049. 21332183 PMC3158846

[41] Carryn S, Van Bambeke F, Mingeot-Leclercq MP, Tulkens PM. 2002. Comparative intracellular (THP-1 macrophage) and extracellular activities of β-lactams, azithromycin, gentamicin, and fluoroquinolones against *Listeria monocytogenes* at clinically relevant concentrations. Antimicrob Agents Chemother 46:2095–2103. doi:10.1128/AAC.46.7.2095-2103.2002. 12069960 PMC127291

[42] Paillard D, Grellet J, Dubois V, Saux M-C, Quentin C. 2002. Discrepancy between uptake and intracellular activity of moxifloxacin in a *Staphylococcus aureus*-human THP-1 monocytic cell model. Antimicrob Agents Chemother 46:288–293. doi:10.1128/AAC.46.2.288-293.2002. 11796332 PMC127041

[43] Barcia-Macay M, Seral C, Mingeot-Leclercq M-P, Tulkens PM, Van Bambeke F. 2006. Pharmacodynamic evaluation of the intracellular activities of antibiotics against *Staphylococcus aureus* in a model of THP-1 macrophages. Antimicrob Agents Chemother 50:841–851. doi:10.1128/AAC.50.3.841-851.2006. 16495241 PMC1426441

[44] Hara T, Takemura H, Kanemitsu K, Yamamoto H, Shimada J. 2000. Comparative uptake of grepafloxacin and ciprofloxacin by a human monocytic cell line, THP-1. J Infect Chemother 6:162–167. doi:10.1007/s101560070016. 11810558

[45] Lemaire S, Van Bambeke F, Tulkens PM. 2009. Cellular accumulation and pharmacodynamic evaluation of the intracellular activity of CEM-101, a novel fluoroketolide, against *Staphylococcus aureus*, *Listeria monocytogenes*, and *Legionella pneumophila* in human THP-1 macrophages. Antimicrob Agents Chemother 53:3734–3743. doi:10.1128/AAC.00203-09. 19564365 PMC2737860

[46] Lemaire S, Van Bambeke F, Mingeot-Leclercq MP, Tulkens PM. 2007. Modulation of the cellular accumulation and intracellular activity of daptomycin towards phagocytized *Staphylococcus aureus* by the P-glycoprotein (MDR1) efflux transporter in human THP-1 macrophages and madin-darby canine kidney cells. Antimicrob Agents Chemother 51:2748–2757. doi:10.1128/AAC.00090-07. 17548493 PMC1932525

[47] Peyrusson F, Tulkens PM, Van Bambeke F. 2018. Cellular pharmacokinetics and intracellular activity of gepotidacin against *Staphylococcus aureus* with different resistance phenotypes in models of cultured phagocytic cells. Antimicrob Agents Chemother 62:e02245-17. doi:10.1128/AAC.02245-17. 29358297 PMC5913948

[48] Van de Velde S, Nguyen HA, Van Bambeke F, Tulkens PM, Grellet J, Dubois V, Quentin C, Saux MC. 2008. Contrasting effects of human THP-1 cell differentiation on levofloxacin and moxifloxacin intracellular accumulation and activity against *Staphylococcus aureus* and *Listeria monocytogenes*. J Antimicrob Chemother 62:518–521. doi:10.1093/jac/dkn232. 18544595

[49] Prideaux B, Via LE, Zimmerman MD, Eum S, Sarathy J, O'Brien P, Chen C, Kaya F, Weiner DM, Chen P-Y, Song T, Lee M, Shim TS, Cho JS, Kim W, Cho SN, Olivier KN, Barry CE, Dartois V. 2015. The association between sterilizing activity and drug distribution into tuberculosis lesions. Nat Med 21:1223–1227. doi:10.1038/nm.3937. 26343800 PMC4598290

[50] Chen C, Gardete S, Jansen RS, Shetty A, Dick T, Rhee KY, Dartois V. 2018. Verapamil targets membrane energetics in *Mycobacterium tuberculosis*. Antimicrob Agents Chemother 62. doi:10.1128/AAC.02107-17. PMC5923092 29463541

[51] Omollo C, Singh V, Kigondu E, Wasuna A, Agarwal P, Moosa A, Ioerger TR, Mizrahi V, Chibale K, Warner DF. 2021. Developing synergistic drug combinations to restore antibiotic sensitivity in drug-resistant *Mycobacterium tuberculosis*. Antimicrob Agents Chemother 65. doi:10.1128/AAC.02554-20. PMC8092878 33619062

[52] Rohde K, Yates RM, Purdy GE, Russell DG. 2007. *Mycobacterium tuberculosis* and the environment within the phagosome. Immunol Rev 219:37–54. doi:10.1111/j.1600-065X.2007.00547.x. 17850480

[53] Vanderven BC, Huang L, Rohde KH, Russell DG. 2016. The minimal unit of infection: *M. tuberculosis* in the macrophage. Microbiol Spectr 4:1–26. doi:10.1128/microbiolspec.TBTB2-0025-2016. PMC5245711 28084213

[54] Zimmerman M, Lestner J, Prideaux B, O'Brien P, Dias-Freedman I, Chen C, Dietzold J, Daudelin I, Kaya F, Blanc L, Chen P-Y, Park S, Salgame P, Sarathy J, Dartois V. 2017. Ethambutol partitioning in tuberculous pulmonary lesions explains its clinical efficacy. Antimicrob Agents Chemother 61. doi:10.1128/AAC.00924-17. PMC5571334 28696241

[55] Tanner L, Denti P, Wiesner L, Warner DF. 2018. Drug permeation and metabolism in *Mycobacterium tuberculosis*: prioritising local exposure as essential criterion in new TB drug development. IUBMB Life 70:926–937. doi:10.1002/iub.1866. 29934964 PMC6129860

[56] Rodvold KA, George JM, Yoo L. 2011. Penetration of anti-infective agents into pulmonary epithelial lining fluid. Clin Pharmacokinet 50:637–664. doi:10.2165/11594090-000000000-00000. 21895037

[57] Rodvold KA, Hope WW, Boyd SE. 2017. Considerations for effect site pharmacokinetics to estimate drug exposure: concentrations of antibiotics in the lung. Curr Opin Pharmacol 36:114–123. doi:10.1016/j.coph.2017.09.019. 29096171

[58] Schuler P, Zemper K, Borner K, Koeppe P, Schaberg T, Lode H. 1997. Penetration of sparfloxacin and ciprofloxacin into alveolar macrophages, epithelial lining fluid, and polymorphonuclear leucocytes. Eur Respir J 10:1130–1136. doi:10.1183/09031936.97.10051130. 9163658

[59] Gotfried MH, Danziger LH, Rodvold KA. 2001. Steady-state plasma and intrapulmonary concentrations of levofloxacin and ciprofloxacin in healthy adult subjects. Chest 119:1114–1122. doi:10.1378/chest.119.4.1114. 11296178

[60] Baldwin DR, Honeybourne D, Wise R. 1992. Pulmonary disposition of antimicrobial agents: in vivo observations and clinical relevance. Antimicrob Agents Chemother 36:1176–1180. doi:10.1128/AAC.36.6.1176. 1416817 PMC190300

[61] Conte JE, Golden JA, McQuitty M, Kipps J, Duncan S, McKenna E, Zurlinden E. 2002. Effects of gender, AIDS, and acetylator status on intrapulmonary concentrations of isoniazid. Antimicrob Agents Chemother 46:2358–2364. doi:10.1128/AAC.46.8.2358-2364.2002. 12121905 PMC127347

[62] Honeybourne D, Tobin C, Jevons G, Andrews J, Wise R. 2003. Intrapulmonary penetration of linezolid. J Antimicrob Chemother 51:1431–1434. doi:10.1093/jac/dkg262. 12746375

[63] Boselli E, Breilh D, Rimmelé T, Djabarouti S, Toutain J, Chassard D, Saux M-C, Allaouchiche B. 2005. Pharmacokinetics and intrapulmonary concentrations of linezolid administered to critically ill patients with ventilator-associated pneumonia. Crit Care Med 33:1529–1533. doi:10.1097/01.ccm.0000168206.59873.80. 16003058

[64] Conte JE, Golden JA, Kipps J, Zurlinden E. 2002. Intrapulmonary pharmacokinetics of linezolid. Antimicrob Agents Chemother 46:1475–1480. doi:10.1128/AAC.46.5.1475-1480.2002. 11959585 PMC127139

[65] Ziglam HM, Baldwin DR, Daniels I, Andrew JM, Finch RG. 2002. Rifampicin concentrations in bronchial mucosa, epithelial lining fluid, alveolar macrophages and serum following a single 600 mg oral dose in patients undergoing fibre-optic bronchoscopy. J Antimicrob Chemother 50:1011–1015. doi:10.1093/jac/dkf214. 12461025

[66] Conte JE, Golden JA, Kipps JE, Lin ET, Zurlinden E. 2004. Effect of sex and AIDS status on the plasma and intrapulmonary pharmacokinetics of rifampicin. Clin Pharmacokinet 43:395–404. doi:10.2165/00003088-200443060-00003. 15086276

[67] Conte JE, Golden JA, McQuitty M, Kipps J, Lin ET, Zurlinden E. 2000. Effects of AIDS and gender on steady-state plasma and intrapulmonary ethionamide concentrations. Antimicrob Agents Chemother 44:1337–1341. doi:10.1128/AAC.44.5.1337-1341.2000. 10770772 PMC89865

[68] Capitano B, Mattoes HM, Shore E, O’Brien A, Braman S, Sutherland C, Nicolau DP. 2004. Steady-state intrapulmonary concentrations of moxifloxacin, levofloxacin, and azithromycin in older adults. Chest 125:965–973. doi:10.1378/chest.125.3.965. 15006955

[69] Conte JE, Golden JA, Duncan S, McKenna E, Zurlinden E. 1999. Intrapulmonary concentrations of pyrazinamide. Antimicrob Agents Chemother 43:1329–1333. doi:10.1128/AAC.43.6.1329. 10348747 PMC89273

[70] Välitalo PAJ, Griffioen K, Rizk ML, Visser SAG, Danhof M, Rao G, van der Graaf PH, van Hasselt JGC. 2016. Structure-based prediction of anti-infective drug concentrations in the human lung epithelial lining fluid. Pharm Res 33:856–867. doi:10.1007/s11095-015-1832-x. 26626793

[71] Aulin LBS, Valitalo PA, Rizk ML, Visser SAG, Rao G, van der Graaf PH, van Hasselt JGC. 2018. Validation of a model predicting anti-infective lung penetration in the epithelial lining fluid of humans. Pharm Res 35:26. doi:10.1007/s11095-017-2336-7. 29368211 PMC5783989

[72] Sarathy JP, Zuccotto F, Hsinpin H, Sandberg L, Via LE, Marriner GA, Masquelin T, Wyatt P, Ray P, Dartois V. 2016. Prediction of drug penetration in tuberculosis lesions. ACS Infect Dis 2:552–563. doi:10.1021/acsinfecdis.6b00051. 27626295 PMC5028112

[73] Lemaire S, Van Bambeke F, Appelbaum PC, Tulkens PM. 2009. Cellular pharmacokinetics and intracellular activity of torezolid (TR-700): studies with human macrophage (THP-1) and endothelial (HUVEC) cell lines. J Antimicrob Chemother 64:1035–1043. doi:10.1093/jac/dkp267. 19759040

[74] Pascual Á, Ballesta S, García I, Perea EJ. 2002. Uptake and intracellular activity of linezolid in human phagocytes and nonphagocytic cells. Antimicrob Agents Chemother 46:4013–4015. doi:10.1128/AAC.46.12.4013-4015.2002. 12435714 PMC132792

[75] Easmon CS, Crane JP. 1985. Uptake of ciprofloxacin by macrophages. J Clin Pathol 38:442–444. doi:10.1136/jcp.38.4.442. 3157704 PMC499174

[76] Carlier M-B, Scorneaux B, Zenebergh A, Desnottes J-F, Tulkens PM. 1990. Cellular uptake, localization and activity of fluoroquinolones in uninfected and infected macrophages. J Antimicrob Chemother 26:27–39. doi:10.1093/jac/26.suppl_B.27. 2258352

[77] Garcia I, Pascual A, Ballesta S, Joyanes P, Perea EJ. 2000. Intracellular penetration and activity of gemifloxacin in human polymorphonuclear leukocytes. Antimicrob Agents Chemother 44:3193–3195. doi:10.1128/AAC.44.11.3193-3195.2000. 11036051 PMC101631

[78] Michot J-M, Seral C, Van Bambeke F, Mingeot-Leclercq M-P, Tulkens PM. 2005. Influence of efflux transporters on the accumulation and efflux of four quinolones (ciprofloxacin, levofloxacin, garenoxacin, and moxifloxacin) in J774 macrophages. Antimicrob Agents Chemother 49:2429–2437. doi:10.1128/AAC.49.6.2429-2437.2005. 15917543 PMC1140503

[79] Irwin SM, Prideaux B, Lyon ER, Zimmerman MD, Brooks EJ, Schrupp CA, Chen C, Reichlen MJ, Asay BC, Voskuil MI, Nuermberger EL, Andries K, Lyons MA, Dartois V, Lenaerts AJ. 2016. Bedaquiline and pyrazinamide treatment responses are affected by pulmonary lesion heterogeneity in *Mycobacterium tuberculosis* infected C3HeB/FeJ Mice. ACS Infect Dis 2:251–267. doi:10.1021/acsinfecdis.5b00127. 27227164 PMC4874602

[80] Lee JM, Park J, Choi S, Jhun BW, Kim S-Y, Jo K-W, Hong JJ, Kim L-H, Shin SJ. 2021. A clofazimine-containing regimen confers improved treatment outcomes in macrophages and in a murine model of chronic progressive pulmonary infection caused by the *Mycobacterium avium* complex. Front Microbiol 11:626216. doi:10.3389/fmicb.2020.626216. 33519787 PMC7841306

[81] Schnappinger D, Ehrt S, Voskuil MI, Liu Y, Mangan JA, Monahan IM, Dolganov G, Efron B, Butcher PD, Nathan C, Schoolnik GK. 2003. Transcriptional adaptation of *mycobacterium tuberculosis* within macrophages: insights into the phagosomal environment. J Exp Med 198:693–704. doi:10.1084/jem.20030846. 12953091 PMC2194186

[82] Mahamed D, Boulle M, Ganga Y, Mc Arthur C, Skroch S, Oom L, Catinas O, Pillay K, Naicker M, Rampersad S, Mathonsi C, Hunter J, Wong EB, Suleman M, Sreejit G, Pym AS, Lustig G, Sigal A. 2017. Intracellular growth of *Mycobacterium tuberculosis* after macrophage cell death leads to serial killing of host cells. Elife 6. doi:10.7554/eLife.28205. PMC5319838 28130921

[83] Cai H, Rose K, Liang LH, Dunham S, Stover C. 2009. Development of a liquid chromatography/mass spectrometry-based drug accumulation assay in *Pseudomonas aeruginosa*. Anal Biochem 385:321–325. doi:10.1016/j.ab.2008.10.041. 19032927

[84] Bhat J, Narayan A, Venkatraman J, Chatterji M. 2013. LC-MS based assay to measure intracellular compound levels in *Mycobacterium smegmatis*: linking compound levels to cellular potency. J Microbiol Methods 94:152–158. doi:10.1016/j.mimet.2013.05.010. 23747411

[85] Bitto NJ, Baker PJ, Dowling JK, Wray-McCann G, De Paoli A, Tran LS, Leung PL, Stacey KJ, Mansell A, Masters SL, Ferrero RL. 2018. Membrane vesicles from *Pseudomonas aeruginosa* activate the non‐canonical inflammasome through caspase‐5 in human monocytes. Immunol Cell Biol 96:1120–1130. doi:10.1111/imcb.12190. 30003588

[86] Sorrentino F, Gonzalez del Rio R, Zheng X, Presa Matilla J, Gomez PT, Hoyos MM, Perez Herran ME, Mendoza Losana A, Av-Gay Y. 2016. Development of an intracellular screen for new compounds able to inhibit *Mycobacterium tuberculosis* growth in human macrophages. Antimicrob Agents Chemother 60:640–645. doi:10.1128/AAC.01920-15. 26503663 PMC4704166

[87] Raffetseder J, Pienaar E, Blomgran R, Eklund D, Patcha Brodin V, Andersson H, Welin A, Lerm M. 2014. Replication rates of *Mycobacterium tuberculosis* in human macrophages do not correlate with mycobacterial antibiotic susceptibility. PLoS One 9:e112426. doi:10.1371/journal.pone.0112426. 25386849 PMC4227709

[88] Lemaire S, Mingeot-Leclercq M-P, Tulkens PM, Van Bambeke F. 2014. Study of macrophage functions in murine J774 cells and human activated THP-1 cells exposed to oritavancin, a lipoglycopeptide with high cellular accumulation. Antimicrob Agents Chemother 58:2059–2066. doi:10.1128/AAC.02475-13. 24449768 PMC4023769

[89] Vallet CM, Marquez B, Ngabirano E, Lemaire S, Mingeot-Leclercq M-P, Tulkens PM, Van Bambeke F. 2018. Cellular accumulation of fluoroquinolones is not predictive of their intracellular activity: studies with gemifloxacin, moxifloxacin and ciprofloxacin in a pharmacokinetic/pharmacodynamic model of uninfected and infected macrophages. Int J Antimicrob Agents 38:249–256. doi:10.1016/j.ijantimicag.2011.05.011. 21764262

[90] Carolina RD, Marisol O, Mary SL, Alfonso PM. 2018. Quantifying intracellular Mycobacterium tuberculosis: an essential issue for in vitro assays. Microbiologyopen 7:e00588. doi:10.1002/mbo3.588. 29484835 PMC5911991

[91] Goutelle S, Bourguignon L, Maire PH, Van Guilder M, Conte JE, Jelliffe RW. 2009. Population modeling and Monte Carlo simulation study of the pharmacokinetics and antituberculosis pharmacodynamics of rifampin in lungs. Antimicrob Agents Chemother 53:2974–2981. doi:10.1128/AAC.01520-08. 19380594 PMC2704682

[92] Soman A, Honeybourne D, Andrews J, Jevons G, Wise R. 1999. Concentrations of moxifloxacin in serum and pulmonary compartments following a single 400 mg oral dose in patients undergoing fibre-optic bronchoscopy. J Antimicrob Chemother 44:835–838. doi:10.1093/jac/44.6.835. 10590288

[93] Breilh D, Jougon J, Djabarouti S, Gordien JB, Xuereb F, Velly JF, Arvis P, Landreau V, Saux MC. 2003. Diffusion of oral and intravenous 400 mg once-daily moxifloxacin into lung tissue at pharmacokinetic steady-state. J Chemother 15:558–562. doi:10.1179/joc.2003.15.6.558. 14998080

[94] Stass H, Kubitza D, Schühly U. 2001. Pharmacokinetics, safety and tolerability of moxifloxacin, a novel 8-methoxyfluoroquinolone, after repeated oral administration. Clin Pharmacokinet 40:1–9. doi:10.2165/00003088-200140001-00001. 11352436

[95] Müller M, Stass H, Brunner M, Möller JG, Lackner E, Eichler HG. 1999. Penetration of moxifloxacin into peripheral compartments in humans. Antimicrob Agents Chemother 43:2345–2349. doi:10.1128/AAC.43.10.2345. 10508004 PMC89480

[96] Gillespie SH. 2016. The role of moxifloxacin in tuberculosis therapy. Eur Respir Rev 25:19–28. doi:10.1183/16000617.0085-2015. 26929417 PMC9487659

[97] van Heeswijk RPG, Dannemann B, Hoetelmans RMW. 2014. Bedaquiline: a review of human pharmacokinetics and drug-drug interactions. J Antimicrob Chemother 69:2310–2318. doi:10.1093/jac/dku171. 24860154

[98] Diacon AH, Donald PR, Pym A, Grobusch M, Patientia RF, Mahanyele R, Bantubani N, Narasimooloo R, De Marez T, Van Heeswijk R, Lounis N, Meyvisch P, Andries K, McNeeley DF. 2012. Randomized pilot trial of eight weeks of bedaquiline (TMC207) treatment for multidrug-resistant tuberculosis: long-term outcome, tolerability, and effect on emergence of drug resistance. Antimicrob Agents Chemother 56:3271–3276. doi:10.1128/AAC.06126-11. 22391540 PMC3370813

[99] Tulkens PM. 1991. Intracellular distribution and activity of antibiotics. Eur J Clin Microbiol Infect Dis 10:100–106. doi:10.1007/BF01964420. 1864271

[100] Silva-Miranda M, Ekaza E, Breiman A, Asehnoune K, Barros-Aguirre D, Pethe K, Ewann F, Brodin P, Ballell-Pages L, Altare F. 2015. High-content screening technology combined with a human granuloma model as a new approach to evaluate the activities of drugs against *Mycobacterium tuberculosis*. Antimicrob Agents Chemother 59:693–697. doi:10.1128/AAC.03705-14. 25348525 PMC4291390

[101] Coertzen D, Reader J, van der Watt M, Nondaba SH, Gibhard L, Wiesner L, Smith P, D'Alessandro S, Taramelli D, Wong HN, Du Preez JL, Wu RWK, Birkholtz L-M, Haynes RK. 2018. Artemisone and artemiside are potent panreactive antimalarial agents that also synergize redox imbalance in *Plasmodium falciparum* transmissible gametocyte stages. Antimicrob Agents Chemother 62. doi:10.1128/AAC.02214-17. PMC6105806 29866868

[102] Gurumurthy M, Rao M, Mukherjee T, Rao SPS, Boshoff HI, Dick T, Barry CE, Manjunatha UH. 2013. A novel F420-dependent anti-oxidant mechanism protects *Mycobacterium tuberculosis* against oxidative stress and bactericidal agents. Mol Microbiol 87:744–755. doi:10.1111/mmi.12127. 23240649 PMC3567243

[103] Sarkar R, Mdladla C, Macingwana L, Pietersen R-D, Ngwane AH, Tabb DL, van Helden PD, Wiid I, Baker B. 2018. Proteomic analysis reveals that sulfamethoxazole induces oxidative stress in *M. tuberculosis*. Tuberculosis (Edinb) 111:78–85. doi:10.1016/j.tube.2018.05.010. 30029919

[104] Ngwane AH, Petersen R-D, Baker B, Wiid I, Wong HN, Haynes RK. 2019. The evaluation of the anti-cancer drug elesclomol that forms a redox-active copper chelate as a potential anti-tubercular drug. IUBMB Life 71:532–538. doi:10.1002/iub.2002. 30698324

[105] National Research Council. 2011. Guide for the care and use of laboratory animals: eighth edition. The National Academies Press, Washington, DC.

[106] Crossley ML, Turner RJ, Hofmann CM, Dreisbach PF, Parker RP. 1952. Chemotherapeutic dyes. II. 5-Arylamino-9-dialkylaminobenzo[a]phenoxazines. J Am Chem Soc 74:578–584. doi:10.1021/ja01123a002.

[107] Stužka V, Šimánek V, Stránský Z. 1967. Infra-red spectroscopy of benzo(α)phenoxazines. Spectrochim Acta Part A Mol Spectrosc 23:2175–2183. doi:10.1016/0584-8539(67)80104-1.

[108] Ge J-F, Arai C, Yang M, Bakar Md A, Lu J, Ismail NSM, Wittlin S, Kaiser M, Brun R, Charman SA, Nguyen T, Morizzi J, Itoh I, Ihara M. 2010. Discovery of novel benzo[a]phenoxazine SSJ-183 as a drug candidate for malaria. ACS Med Chem Lett 1:360–364. doi:10.1021/ml100120a. 24900219 PMC4007839

[109] Shi X-L, Ge J-F, Liu B-Q, Kaiser M, Wittlin S, Brun R, Ihara M. 2011. Synthesis and in vitro antiprotozoal activities of 5-phenyliminobenzo[a]phenoxazine derivatives. Bioorg Med Chem Lett 21:5804–5807. doi:10.1016/j.bmcl.2011.07.112. 21868222

[110] Akladios FN, Andrew SD, Parkinson CJ. 2015. Selective induction of oxidative stress in cancer cells via synergistic combinations of agents targeting redox homeostasis. Bioorg Med Chem 23:3097–3104. doi:10.1016/j.bmc.2015.05.006. 26022081

[111] Crossley ML, Hofmann CM, Dreisbach PF. 1952. Chemotherapeutic dyes. III. 5-Heterocyclicamino-9-dialkylaminobenzo[a]phenoxazines. J Am Chem Soc 74:584–586. doi:10.1021/ja01123a003.

[112] Beteck RM, Seldon R, Coertzen D, van der Watt ME, Reader J, Mackenzie JS, Lamprecht DA, Abraham M, Eribez K, Müller J, Rui F, Zhu G, de Grano RV, Williams ID, Smit FJ, Steyn AJC, Winzeler EA, Hemphill A, Birkholtz L-M, Warner DF, N’Da DD, Haynes RK. 2018. Accessible and distinct decoquinate derivatives active against *Mycobacterium tuberculosis* and apicomplexan parasites. Commun Chem 1:62. doi:10.1038/s42004-018-0062-7.

[113] Mosmann T. 1983. Rapid colorimetric assay for cellular growth and survival: application to proliferation and cytotoxicity assay. J Immunol Methods 65:55–63. doi:10.1016/0022-1759(83)90303-4. 6606682

[114] Wood R, Morrow C, Barry IIC, Bryden WA, Call CJ, Hickey AJ, Rodes CE, Scriba TJ, Blackburn J, Issarow C, Mulder N, Woodward J, Moosa A, Singh V, Mizrahi V, Warner DF. 2016. Real-time investigation of tuberculosis transmission: developing the respiratory aerosol sampling chamber (RASC). PLoS One 11:e0146658. doi:10.1371/journal.pone.0146658. 26807816 PMC4726558

[115] Chan K, Knaak T, Satkamp L, Humbert O, Falkow S, Ramakrishnan L. 2002. Complex pattern of *Mycobacterium marinum* gene expression during long-term granulomatous infection. Proc Natl Acad Sci USA 99:3920–3925. doi:10.1073/pnas.002024599. 11891270 PMC122624

[116] Chen P, Gearhart J, Protopopova M, Einck L, Nacy CA. 2006. Synergistic interactions of SQ109, a new ethylene diamine, with front-line antitubercular drugs in vitro. J Antimicrob Chemother 58:332–337. doi:10.1093/jac/dkl227. 16751637

[117] Odds FC. 2003. Synergy, antagonism, and what the chequerboard puts between them. J Antimicrob Chemother 52:1. doi:10.1093/jac/dkg301. 12805255

[118] Tanner L, Evans JC, Seldon R, Jordaan A, Warner DF, Haynes RK, Parkinson CJ, Wiesner L. 2019. In vitro efficacies, ADME, and pharmacokinetic properties of phenoxazine derivatives active against *Mycobacterium tuberculosis*. Antimicrob Agents Chemother 63:e01010-19. doi:10.1128/AAC.01010-19. 31427302 PMC6811447

