# Supplementary material for: Intracellular Accumulation of Novel and Clinically Used TB Drugs Potentiates Intracellular Synergy
Source: Microbiol Spectr. 2021 Sep 29;9(2):e00434-21. doi: 10.1128/Spectrum.00434-21 (PMC8557888; doi:10.1128/Spectrum.00434-21)
Supplement: SUPPLEMENTAL FILE 1 — Supplemental material. Download SPECTRUM00434-21_Supp_1_seq10.pdf, PDF file, 0.5 MB [file spectrum00434-21_supp_1_seq10.pdf]

Intracellular accumulation of novel and clinically used TB drugs potentiates intracellular synergy

Running title: ***Intracellular accumulation and efficacies of novel TB active compounds***

Lloyd Tanner[a#], Gabriel T. Mashabela[b], Charles C. Omollo[b], Timothy J. de Wet[b], Christopher J. Parkinson[c], Digby F. Warner[b,d], Richard K. Haynes[e], Lubbe Wiesner[a]

a. Division of Clinical Pharmacology, Department of Medicine, University of Cape Town, Observatory, South Africa.
b. SAMRC/NHLS/UCT Molecular Mycobacteriology Research Unit, DST/NRF Centre of Excellence for Biomedical TB Research, Department of Pathology and Institute of Infectious Disease and Molecular Medicine, Faculty of Health Sciences, University of Cape Town, South Africa
c. School of Biomedical Sciences, Charles Sturt University, Orange, New South Wales, Australia.
d. Wellcome Centre for Infectious Diseases Research in Africa, University of Cape Town, South Africa.
e. Centre of Excellence for Pharmaceutical Sciences, Faculty of Health Sciences, North-West University, Potchefstroom, South Africa

Correspondence:

#Address correspondence to: Lloyd Tanner, lloyd.tanner@med.lu.se

Supplementary figures and tables:

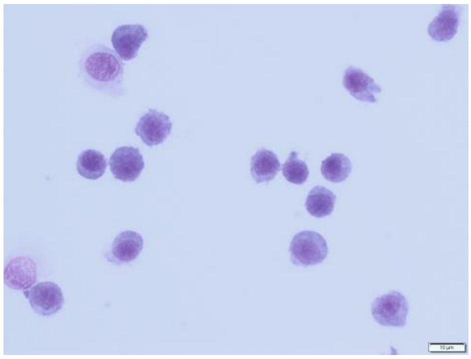

Figure S1: Murine alveolar macrophage cells treated with PhX1 retrieved from BALF sampling. Light microscopy (40X) of murine cells from BALF sampling, demonstrating the predominance of alveolar macrophages.

Table S1: MS/MS settings

Analyte	Transition (m/z)	Dwell time (ms)	Declustering potential (V)	Entrance potential (V)	Collison energy (eV)	Cell exit potential (V)
RMB041	505.2→418.0	150	81	10	43	10
WHN296	489.2→223.2	150	86	10	55	18
PhX1	395.2→351.2	150	66	10	49	22
PhX2	428.93→370.4	150	68	10	54	21
PhX6	395.47→341.46	150	76	10	59	12
PhX8	395.48→351.60	150	66	10	49	22
PhX10	396.46→352.40	150	72	10	48	21
PhX14	408.52→350.20	150	76	10	41	24
PhX15	424.52→351.9	150	68	10	44	20

Carbamaz-epine (IS)	237.1→194.1	150	71	10	29	14
BDQ	555.21→57.8	150	26	10	31	14
MXF	402.19→384.0	150	52	10	33	24
RIF	823.91→791.2	150	46	10	25	14
LNZ	337.86→296.30	150	51	10	27	18
LVX	362.40→318.10	150	53	10	29	16
CLZ	474.4→431.31	150	42	10	22	12

Table S2: Electrospray ionization settings

Analyte	Ion spray voltage (V)	Nebulizer gas (AU)	Curtain gas (AU)	Turbo gas (AU)	Source temperature (°C)
RMB041	4500	40	20	20	400
WHN296	4500	50	25	25	400
PhX1; PhX 2; PhX6; PhX8; PhX 10; PhX14; PhX15; Carbamaz-epine	4500	40	20	20	400
BDQ	4000	40	20	20	400
MXF	4500	40	25	25	400
RIF	4000	30	10	10	400
	4500	45	15	15	400

LNZ					
LVX	4500	40	20	20	400
CLZ	4500	40	20	20	400

Table S3: LC elution conditions

<table>
<tr><th>Analyte</th><th>Column specifications</th><th>Mobile Phase A</th><th>Mobile Phase B</th><th>Flow rate (µL/min)</th><th>Gradient profile</th></tr>
<tr><td>RMB041</td><td>Gemini-NX, 5µm, C18, 50 x 2.0 mm</td><td>0.1% FA in $H_2$0</td><td>0.1% FA in ACN</td><td>300</td><td><table>
<tr><th>Time (min)</th><th>%A</th><th>%B</th></tr>
<tr><td>0.25</td><td>90</td><td>10</td></tr>
<tr><td>2.75</td><td>10</td><td>900</td></tr>
<tr><td>3.4</td><td>10</td><td>90</td></tr>
<tr><td>3.45</td><td>90</td><td>10</td></tr>
<tr><td>8</td><td>90</td><td>10</td></tr>
</table></td></tr>
<tr><td>WHN296</td><td>Gemini-NX, 5µm, C18, 50 x 2.0 mm</td><td>0.1% FA in $H_2$0</td><td>0.1% FA in ACN</td><td>300</td><td><table>
<tr><th>Time (min)</th><th>%A</th><th>%B</th></tr>
<tr><td>0.25</td><td>95</td><td>5</td></tr>
<tr><td>2.0</td><td>5</td><td>95</td></tr>
<tr><td>3.4</td><td>5</td><td>95</td></tr>
<tr><td>3.45</td><td>95</td><td>5</td></tr>
<tr><td>6</td><td>95</td><td>5</td></tr>
</table></td></tr>
<tr><td>PhX1; PhX2; PhX6; PhX8; PhX 10; PhX14; PhX 15; Carbamazepine</td><td>Gemini-NX, 5µm, C18, 50 x 2.0 mm</td><td>0.1% FA in $H_2$0</td><td>0.1% FA in ACN</td><td>400</td><td><table>
<tr><th>Time (min)</th><th>%A</th><th>%B</th></tr>
<tr><td>0.25</td><td>90</td><td>10</td></tr>
<tr><td>1.75</td><td>10</td><td>900</td></tr>
<tr><td>4.7</td><td>10</td><td>90</td></tr>
<tr><td>4.75</td><td>90</td><td>10</td></tr>
<tr><td>8</td><td>90</td><td>10</td></tr>
</table></td></tr>
<tr><td>BDQ</td><td>Atlantis T3; 5µm; C18; 100 x 2.1 mm</td><td>0.1% FA in $H_2$0</td><td>0.1% FA in ACN</td><td>300</td><td><table>
<tr><th>Time (min)</th><th>%A</th><th>%B</th></tr>
<tr><td>0.25</td><td>60</td><td>40</td></tr>
<tr><td>1.75</td><td>0</td><td>100</td></tr>
<tr><td>2.7</td><td>0</td><td>100</td></tr>
<tr><td>2.75</td><td>60</td><td>40</td></tr>
<tr><td>6</td><td>60</td><td>40</td></tr>
</table></td></tr>
</table>

<table>
<tr><td>MXF</td><td>Gemini-NX, 5µm, C18, 50 x 2.0 mm</td><td>0.1% FA in H_20</td><td>0.1% FA in ACN</td><td>400</td><td><table><tr><th>Time (min)</th><th>%A</th><th>%B</th></tr><tr><td>0.25</td><td>90</td><td>10</td></tr><tr><td>2.75</td><td>10</td><td>900</td></tr><tr><td>3.4</td><td>10</td><td>90</td></tr><tr><td>3.45</td><td>90</td><td>10</td></tr></table></td></tr>
<tr><td>RIF</td><td>Discovery, 5µm, C18, 50 x 4.6 mm</td><td>0.1% FA in H_20</td><td>0.1% FA in MeOH</td><td>300</td><td>Isocratic conditions (60:40)</td></tr>
<tr><td>LNZ</td><td>Poroshell 120EC-C184.6 X 50mm, 2.7 um</td><td>0.1% FA in H_20</td><td>0.1 % FA in MeOH</td><td>300</td><td>Isocratic conditions (60:40)</td></tr>
<tr><td>LVX</td><td>Gemini-NX, 5µm, C18, 50 x 2.0 mm</td><td>0.1% FA in H_20</td><td>0.1% FA in ACN</td><td>400</td><td><table><tr><th>Time (min)</th><th>%A</th><th>%B</th></tr><tr><td>0.25</td><td>90</td><td>10</td></tr><tr><td>2.75</td><td>10</td><td>900</td></tr><tr><td>3.4</td><td>10</td><td>90</td></tr><tr><td>3.45</td><td>90</td><td>10</td></tr><tr><td>8</td><td>90</td><td>10</td></tr></table></td></tr>
<tr><td>CLZ</td><td>Gemini-NX, 5µm, C18, 50 x 2.0 mm</td><td>0.1% FA in H_20</td><td>0.1% FA in ACN</td><td></td><td><table><tr><th>Time (min)</th><th>%A</th><th>%B</th></tr><tr><td>0.25</td><td>90</td><td>10</td></tr><tr><td>2.75</td><td>10</td><td>900</td></tr><tr><td>3.4</td><td>10</td><td>90</td></tr><tr><td>3.45</td><td>90</td><td>10</td></tr><tr><td>8</td><td>90</td><td>10</td></tr></table></td></tr>
</table>
